# Edible and Medicinal Mushrooms in Metabolic Syndrome: From Mechanistic Plausibility to Clinical Evidence—A Narrative Review

**DOI:** 10.3390/nu18213646

**Published:** 2026-11-02

**Authors:** Klaudia Melkis, Karolina Wrońska, Natalia Antoniewska, Joanna Walczyńska, Amelia Kostrzewa, Kinga Szymczykowska, Karolina Jakubczyk

**Affiliations:** Department of Human Nutrition and Metabolomics, Pomeranian Medical University in Szczecin, 24 Broniewskiego Street, 71-460 Szczecin, Poland; klaudia.melkis@pum.edu.pl (K.M.); 74985@student.pum.edu.pl (K.W.); kinga.szymczykowska@pum.edu.pl (K.S.)

**Keywords:** metabolic syndrome, medicinal mushrooms, edible mushrooms, *Ganoderma lucidum*, insulin resistance, dyslipidemia, oxidative stress, gut microbiota, clinical evidence

## Abstract

Background: Edible and medicinal mushrooms contain bioactive compounds potentially relevant to metabolic syndrome (MetS). Methods: This narrative review was based on searches of PubMed and Embase from database inception to 10 April 2026. In vitro, in silico, animal and human studies, and systematic reviews were considered. Evidence was mapped by species, preparation, evidence level and outcome, distinguishing MetS diagnostic components from related outcomes. Results: The evidence was predominantly preclinical and addressed glucose and lipid metabolism, adiposity, inflammation, oxidative stress and gut microbiota. Human evidence was dominated by *Ganoderma lucidum*. The only randomized trial in participants with diagnosed MetS that assessed its diagnostic components (*n* = 84) found no effect of *G. lucidum*, alone or combined with *Cordyceps sinensis*, on glycemia, blood pressure, lipids, waist circumference or MetS status. Systematic reviews reported no significant effects on fasting blood glucose, blood pressure or lipid profile and a small reduction in body mass index without a corresponding change in waist circumference, although the certainty of evidence was very low. Individual, unreplicated trials reported improvements in specific components. Conclusions: Mechanistic plausibility has not translated into demonstrated clinical efficacy. Insufficient chemical characterization and standardization of preparations preclude species-specific recommendations. Trials of standardized preparations in participants with diagnosed MetS are needed.

## 1. Introduction

Metabolic syndrome (MetS) is characterized by a cluster of cardiometabolic abnormalities, including abdominal obesity, hypertension, dyslipidemia and impaired glucose regulation, and is associated with an increased risk of cardiovascular disease, type 2 diabetes and other chronic diseases [1]. Its prevalence varies considerably across geographic regions, affecting more than 20% of adults in the United States, approximately 25% in Europe and 24.2% in China [2,3]. Recent data indicate that the global prevalence of MetS increased from 11.9% in 2000 to 29.4% in 2023, corresponding to approximately 1.54 billion affected adults worldwide [4]. According to the harmonized definition, MetS is diagnosed when at least three of five criteria are met: elevated waist circumference according to population- and country-specific thresholds, triglycerides ≥ 1.7 mmol/L (150 mg/dL), high-density lipoprotein cholesterol (HDL-C) < 1.0 mmol/L (40 mg/dL) in men or <1.3 mmol/L (50 mg/dL) in women, systolic blood pressure ≥ 130 mmHg and/or diastolic blood pressure ≥ 85 mmHg, and fasting glucose ≥ 5.6 mmol/L (100 mg/dL), with treatment for each respective abnormality also satisfying the corresponding criterion [5]. In this review, these five criteria are referred to as diagnostic components, whereas insulin resistance, body mass index (BMI), total and low-density lipoprotein cholesterol (LDL-C), inflammatory and oxidative stress markers and gut microbiota composition are treated as related outcomes.

Lifestyle modification plays a key role in the prevention and treatment of metabolic syndrome. Evidence indicates that an appropriately balanced diet tailored to individual needs, together with regular physical activity, forms the basis of effective MetS prevention [6]. Dietary interventions may contribute not only to weight reduction but also to improvements in lipid profiles, insulin sensitivity and glycemic control [7]. Interest has also grown in the role of functional foods and nutraceuticals as adjuncts to the prevention and treatment of metabolic diseases [8]. Particular attention has been paid to natural sources of bioactive compounds, which are attracting increasing interest among patients, researchers and clinicians. Among these sources are edible and medicinal mushrooms, which contain diverse compounds with potential health benefits [9].

Mushrooms have been used in cuisines around the world for centuries and are valued both for their sensory properties, including taste and texture, and for their use in traditional medicine. To date, approximately 150,000 fungal species have been described, whereas the total number is estimated at 2.2–3.8 million. Of these, approximately 2000 are considered edible, and more than 200 exhibit properties with medicinal potential [10]. The distinction between edible and medicinal mushrooms is primarily based on their traditional and contemporary uses rather than on a formal taxonomic classification. Edible mushrooms are commonly consumed as foods, whereas medicinal mushrooms are frequently marketed as extracts, powders or dietary supplements, with considerable variation in their chemical characterization and degree of standardization [11]. Among the most widely studied fungal materials are *Ganoderma* spp., *Cordyceps militaris*, *Ophiocordyceps sinensis* (formerly *Cordyceps sinensis*) [12], *Pleurotus ostreatus*, *Lentinula edodes*, and *Hericium erinaceus*. Their biological activity is associated with the presence of numerous bioactive compounds, such as polysaccharides, terpenoids and phenolic compounds, which have been investigated for antioxidant, anti-inflammatory and immunomodulatory effects and for their potential influence on metabolic processes, including regulation of glucose and lipid metabolism, body weight and cardiovascular function [11,13].

Despite increasing research on edible and medicinal mushrooms, the evidence remains scattered and heterogeneous. Previous reviews have addressed the potential metabolic effects of edible and medicinal mushrooms, including their relevance to MetS and related conditions [14,15]. Other syntheses have focused on *Pleurotus species* [16,17], the association between mushroom consumption and cardiometabolic outcomes [18], or the clinical effects of *Ganoderma lucidum* supplementation [19]. Building on these contributions, the present review integrates evidence across multiple fungal taxa and distinct preparations within a framework explicitly anchored in the diagnostic criteria of MetS. Its incremental contribution is to distinguish diagnostic components from related surrogate and mechanistic outcomes, classify human intervention studies according to their directness to MetS, and critically assess the translational relevance of experimental models, doses and preparation characteristics. This approach identifies specific discontinuities between mechanistic plausibility and demonstrated clinical effects and provides a basis for prioritizing future clinical investigations.

Accordingly, this review aimed to provide a critical narrative synthesis of mechanistic, preclinical and clinical evidence on edible and medicinal mushrooms in relation to MetS. Specifically, it sought to distinguish formal MetS diagnostic components from related mechanistic outcomes, to classify human studies according to their relevance to MetS, to map the evidence by species, preparation and level of evidence, and to appraise the translational relevance of preclinical findings, and thereby identify gaps in species-specific and clinical evidence.

## 2. Methods

### 2.1. Literature Search and Study Selection

This narrative review was prepared with reference to the SANRA criteria [20]. PubMed and Embase were searched from their inception through 10 April 2026. The search strategy combined terms related to edible and medicinal mushrooms, relevant fungal taxa, mushroom-derived preparations and isolated fungal compounds with terms related to metabolic syndrome, its diagnostic components and related mechanistic outcomes, including glucose metabolism, insulin resistance, dyslipidemia, obesity, blood pressure, inflammation, oxidative stress and gut microbiota (Supplementary Table S6). Searches were conducted separately for each thematic area and supplemented by targeted searches for human intervention studies and systematic reviews. Reference lists of relevant publications were also screened manually to identify additional sources. Publications were initially screened by the authors responsible for each thematic area, and potentially relevant studies were subsequently discussed within the author team. Final inclusion decisions were reached by consensus.

### 2.2. Eligibility Criteria

Publications were eligible if they investigated edible or medicinal mushrooms, fungal strains, mushroom-derived preparations, extracts or isolated fungal compounds and reported outcomes related to metabolic syndrome, its diagnostic components or related mechanisms. In vitro, in silico and animal studies were considered; human evidence was restricted to controlled intervention studies in adults. Systematic reviews and meta-analyses synthesizing relevant human intervention studies were included as secondary clinical evidence, including reviews that also incorporated observational studies. Observational findings were not treated as primary interventional evidence in the present review. Conference abstracts and non-English publications were excluded. Primary trials already pooled in an included meta-analysis were presented individually only when they provided information not captured by the pooled estimates, such as enrollment of participants with diagnosed MetS or data on a specific preparation.

### 2.3. Data Extraction and Evidence Synthesis

The following information was extracted from eligible studies: fungal species or strain, tested material or preparation, experimental model, study design, dose or concentration, intervention duration and outcomes. Where available, information on the fungal part used, extraction procedure, chemical characterization and standardization of the preparation was also recorded. Human outcomes were classified either as MetS diagnostic components according to the harmonized definition [5] or as related outcomes, and human studies were categorized as providing direct, component-level or indirect evidence. Findings were synthesized qualitatively and mapped by fungal species, preparation, evidence level and MetS-related outcome to identify areas with concentrated evidence, areas supported mainly by preclinical studies and clinically understudied species.

### 2.4. Methodological Appraisal

The methodological quality of human trials was described using design features relevant to risk of bias, including randomization, blinding, placebo control, trial registration, sample size and analysis population, and considered alongside the risk-of-bias and evidence-certainty assessments reported in the included systematic reviews [19,21]. The translational relevance of preclinical studies was assessed with respect to the experimental model, study design, dose and chemical characterization of the preparation. Selected doses reported in animal studies were converted into human-equivalent doses using body surface area normalization [22].

## 3. Bioactive Components of Mushrooms

Mushrooms are a rich source of diverse bioactive compounds, whose composition and concentrations vary across species and according to cultivation conditions, the part of the mushroom used, such as the fruiting body or mycelium, and the extraction method applied [23]. Despite this variability, several major groups can be distinguished in many edible and medicinal species, including polysaccharides, triterpenes, antioxidant compounds and other substances, such as sterols and nucleosides. This variability is important when interpreting biological effects because preparations obtained from the same species may differ substantially in composition.

### 3.1. Polysaccharides: β-Glucans and Related Compounds

Polysaccharides, mainly β-glucans and heteropolysaccharides, are structural components of the fungal cell wall and are among the best-characterized bioactive constituents of mushrooms. The biological activity of β-glucans depends on their spatial structure, type of glycosidic linkages, branching and solubility. They have been reported to exert immunomodulatory effects by activating macrophages and natural killer (NK) cells and increasing cytokine production [24], and highly soluble β-glucans have been shown to be more effective in modulating inflammatory cytokines [25]. Their potential antiglycemic, hypolipidemic and cardioprotective effects [26] have been attributed to the inhibition of carbohydrate-digesting enzymes, such as α-amylase and α-glucosidase [27], to improved insulin sensitivity, protection of pancreatic β-cells and modulation of PI3K/Akt signaling [28], and to prebiotic effects, including the stimulation of beneficial gut bacteria and short-chain fatty acid production [29].

In an analysis of cultivated and wild mushrooms, β-glucan content ranged from 3.79% of dry matter in a *Cordyceps*-related sample to 60.79% in turkey tail (*Trametes versicolor*) [30]. Shiitake (*Lentinula edodes*) is a particularly valuable source of lentinan, a β-glucan with immunomodulatory properties [31], whereas heteropolysaccharides and polysaccharide–protein complexes of reishi (*Ganoderma lucidum*) have attracted attention for their anti-inflammatory and antioxidant activity and possible synergy with β-glucans [32].

### 3.2. Triterpenes and Terpenoid Compounds

Triterpenes, secondary metabolites composed of isoprene units [33], exhibit anti-inflammatory, hepatoprotective and antioxidant activities [34]. Preclinical studies also suggest their involvement in the regulation of glucose and lipid metabolism through reduced oxidative stress, inhibition of inflammatory mediators and modulation of NF-κB and MAPK signaling [35]. *G. lucidum* is a particularly rich source of these compounds, with 86 triterpenoids, including ganoderic, lucidenic and ganolucidic acids, ganoderiols and ganosporelactones, identified in its fruiting bodies by untargeted metabolomics [36]. Their proposed relevance to metabolic disorders involves effects on oxidative stress, inflammatory pathways, gut microbiota and cholesterol metabolism [37].

### 3.3. Antioxidant Compounds

Mushrooms also contain numerous compounds with antioxidant properties, including polyphenols, polysaccharides, carotenoids, ergosterol and its derivatives and thiols such as ergothioneine and glutathione [38]. These compounds may act directly by scavenging reactive oxygen and nitrogen species and inhibiting lipid peroxidation, and indirectly by supporting antioxidant enzymes such as superoxide dismutase, catalase and glutathione peroxidase [39]. The phenolic profile of edible mushrooms is dominated by hydroxybenzoic and hydroxycinnamic acid derivatives, such as gallic, protocatechuic, caffeic and ferulic acids, although their presence and concentration depend on the species and the analytical method [40,41].

Ergothioneine has attracted particular attention, a water-soluble thiol derivative of histidine for which edible mushrooms are among the main dietary sources [38]. In addition to neutralizing reactive oxygen species and chelating metal ions, ergothioneine is taken up by the specific transporter OCTN1, which allows its accumulation in tissues exposed to oxidative stress [42]. A comparison of five *Pleurotus* species illustrates how these constituents combine. The methanolic extract of *P. flabellatus*, rich in ergothioneine, ergosterol and mannitol, showed the strongest radical-scavenging activity, whereas that of *P. ostreatus*, with the highest β-glucan content, most strongly inhibited NF-κB/AP-1 activity, suggesting that the activity of mushroom extracts may reflect multiple, potentially interacting components [43].

### 3.4. Other Bioactive Compounds

Mushrooms also provide other bioactive substances, including sterols, nucleosides, lectins, peptides and immunomodulatory proteins. Ergosterol, the main fungal sterol, is a precursor of vitamin D_2_ and can be converted to this vitamin upon exposure to ultraviolet radiation. It is also being investigated for anti-inflammatory, antioxidant and immunomodulatory effects [44,45]. Eritadenine, a nitrogen-containing compound present in shiitake, has hypocholesterolemic activity attributed to effects on hepatic cholesterol and phospholipid metabolism, although this mechanism is documented mainly in experimental studies [46].

Among nucleosides, cordycepin (3′-deoxyadenosine), an adenosine analog characteristic of *Cordyceps militaris*, has been reported to exert antidiabetic, antihyperlipidemic, anti-inflammatory, antioxidant and cardioprotective effects [47]. In a mouse model of type 2 diabetes, *C. militaris* extract and cordycepin improved selected parameters of glucose and lipid metabolism, reduced oxidative stress and inflammation and modulated the gut microbiota and its metabolites [48]. Mushroom-derived peptides may also contribute to metabolic and cardiovascular effects through antioxidant, anti-inflammatory and antihypertensive activity, particularly through inhibition of angiotensin-converting enzyme [49].

Edible and medicinal mushrooms contain a complex mixture of bioactive compounds, including polysaccharides, triterpenoids, antioxidant compounds, sterols, nucleosides and peptides. Their potential metabolic relevance is unlikely to result from a single compound class, but rather from the combined and possibly interacting effects of multiple constituents. This complexity may partly explain the range of biological effects observed in experimental studies, but also makes it difficult to attribute specific metabolic outcomes to individual molecules.

## 4. Effects of Medicinal and Edible Mushrooms on the Components of Metabolic Syndrome

### 4.1. Carbohydrate Metabolism and Insulin Resistance

Disturbances in carbohydrate metabolism and insulin resistance are central features of metabolic syndrome [50]. Reduced insulin sensitivity impairs glucose uptake in skeletal muscle and adipose tissue, enhances hepatic gluconeogenesis and promotes compensatory hyperinsulinemia, potentially contributing to persistent hyperglycemia. In a systematic review and meta-analysis of the adult population, the pooled prevalence of insulin resistance was estimated at 26.53%, with substantial heterogeneity partly attributable to differences in study populations and HOMA-IR cut-off values [51]. According to the harmonized definition, elevated fasting glucose (≥5.6 mmol/L) or drug treatment for elevated glucose fulfills the glycemic criterion for MetS, whereas insulin resistance itself is not a diagnostic criterion [5].

In adipose tissue, reduced insulin responsiveness limits the antilipolytic action of insulin, leading to increased release of free fatty acids (FFAs). Elevated FFA concentrations can exacerbate insulin resistance in the liver and skeletal muscle, promote ectopic lipid accumulation and disrupt insulin signaling. Increased FFA influx into the liver may also enhance very-low-density lipoprotein (VLDL) production, contributing to hypertriglyceridemia, atherogenic dyslipidemia and cardiovascular risk [1,52].

At the molecular level, insulin resistance is associated with impaired insulin receptor signaling and alterations downstream of the receptor. Increased serine/threonine phosphorylation of IRS-1, induced in part by pro-inflammatory cytokines such as TNF-α, may weaken insulin signal transduction [53]. In skeletal muscle, impaired insulin-stimulated GLUT4 translocation limits glucose uptake and utilization [54]. These disturbances are closely linked to chronic low-grade inflammation, oxidative stress and endothelial dysfunction, thereby promoting the progression of MetS and its cardiovascular complications [2].

#### 4.1.1. *Ganoderma* spp.

An in vitro study in HepG2 cells evaluated *Ganoderma lucidum* in a model of palmitic acid (PA)-induced metabolic dysfunction and insulin resistance. An ethanolic extract of *G. lucidum* (20–80 μg/mL) increased cellular glucose consumption and reduced processes associated with gluconeogenesis [55]. In the same study, network pharmacology and molecular docking predicted that β-sitosterol, one of the constituents of the extract, binds to CASP3, PRKACA and PGR. In separate experiments, treatment with the whole extract decreased the mRNA expression of CASP3, PRKACA, CYP19A1, NR3C1 and JUN and increased that of PGR and RXRA (Supplementary Table S1) [55]. Because β-sitosterol was not tested independently and predicted binding does not establish changes in gene expression, the contribution of this compound to the observed changes remains hypothetical. In vivo, a *G. lucidum* proteoglycan (FYGL) lowered fasting glucose and an insulin resistance index in ob/ob mice and enhanced glucose uptake in L6 myotubes through the IRS1/PI3K/Akt/AMPK/GLUT4 axis [56]. In a study by Petryn et al., a lyophilized mycelial extract of *G. lucidum* given to rats with fructose-induced MetS reduced the glucose area under the curve after glucose loading and the fructosamine concentration, and inhibited α-amylase and α-glucosidase in vitro (Supplementary Table S1) [57]. This was one of the few preclinical studies using a model specifically designed to reproduce MetS. Together, these in vitro and animal findings suggest that specific *G. lucidum* preparations may modulate pathways involved in carbohydrate metabolism and insulin resistance.

However, these observations have not been shown to translate into clinical efficacy. The only randomized trial that enrolled participants with formally diagnosed MetS and assessed its diagnostic components (NCEP-ATP III criteria, all with type 2 diabetes, *n* = 84) found that *G. lucidum* (3 g/day for 16 weeks), alone or combined with *Cordyceps sinensis*, did not affect HbA1c (glycated hemoglobin) or fasting plasma glucose and did not reduce the number of participants meeting MetS criteria (Table 1) [58]. Consistently, systematic reviews found no significant effect of *G. lucidum* on glycemic outcomes [19,21,59]. In the most recent meta-analysis, which included 17 randomized clinical trials with 971 participants, six of them testing multi-ingredient products, the certainty of evidence was rated very low for all assessed outcomes using the GRADE approach, supporting the need for further well-designed clinical studies [19].

#### 4.1.2. *Cordyceps* and *Ophiocordyceps* spp.

Selenium-enriched *Cordyceps militaris* polysaccharides obtained by biotransformation (Se-CMP) altered glucose metabolism in an in vitro model of insulin resistance. Specifically, they increased glucose uptake and glycogen synthesis in HepG2 cells. Chen et al. demonstrated that Se-CMP alleviated insulin resistance-induced disturbances in glucose metabolism, among other mechanisms by increasing the expression and translocation of the GLUT4 transporter, which plays an important role in cellular glucose uptake [70]. These effects were obtained in a hepatoma cell line at concentrations of up to 1000 μg/mL and require confirmation in vivo.

In another study, polysaccharides isolated from *C. militaris* (CM-P) were administered to diabetic mice. CM-P supplementation reduced blood glucose and insulin concentrations and improved insulin sensitivity and glucose tolerance, with the effect depending on the dose used (Supplementary Table S1). The strongest effect was observed after administration of CM-P at a dose of 800 mg/kg for four weeks. In this group, blood glucose concentration decreased by 26.69% compared with the diabetic control group. The authors also demonstrated improved insulin sensitivity following CM-P administration, further indicating their potential significance in the regulation of carbohydrate metabolism [71]. The most effective dose of CM-P corresponds to a human-equivalent dose of approximately 3.9 g/day of purified polysaccharide for a 60 kg adult (Table 2). In vitro, CM-P inhibited α-amylase and α-glucosidase by up to 63.82% and 60.81%, respectively, within the tested range of 1.0–10.0 mg/mL [71]. Whether such concentrations are reached in the intestinal lumen after dietary intake is unknown.

Evidence is also available for a fermented preparation identified as *Cordyceps sinensis* (fermented CS) in an in vitro model relevant to diabetic kidney disease (Supplementary Table S1). In high-glucose-treated HK-2 cells, fermented CS (50 μg/mL) attenuated cytotoxicity and apoptosis and promoted cell proliferation (Supplementary Table S1) [72].

**Table 2:** Human-equivalent doses (HED) of mushroom preparations in the in vivo studies summarized in Supplementary Tables S1–S5, estimated by body surface area normalization for a 60 kg adult [22]. Doses in human trials ranged from 0.54 to 10.4 g/day (Table 1). The values are approximate benchmarks and do not account for differences in absorption, particularly of poorly absorbed polysaccharides acting within the gut.

Preparation [Ref]	Model	Effective Dose in Animals	HED (mg/kg)	Approximate Dose for a 60 kg Adult
*G. lucidum* ethanol extract [73]	Mouse	100–200 mg/kg/day	8.1–16.2	0.5–1.0 g/day
*G. lucidum* spore polysaccharide [74]	Mouse, HFD	100–300 mg/kg/day	8.1–24.3	0.5–1.5 g/day
*G. lucidum* sporoderm-broken spore powder [75]	Mouse, HFD	650 mg/kg/day	52.7	3.2 g/day
*G. lucidum* triterpenoid fraction [76]	Mouse, HFD	100–200 mg/kg/day	8.1–16.2	0.5–1.0 g/day
*G. lucidum* proteoglycan FYGL [56]	ob/ob mouse	150–400 mg/kg/day	12.2–32.4	0.7–1.9 g/day
*G. lucidum* mycelial extract [57]	Rat, fructose-induced MetS	1000 mg/kg/day	162	9.7 g/day
GMD, synthetic ganomycin I derivative [77]	ApoE−/− mouse, HFD	5–10 mg/kg/day	0.4–0.8	0.02–0.05 g/day
*C. militaris* polysaccharide CM-P [71]	Mouse, diabetes	800 mg/kg/day	64.9	3.9 g/day
*C. militaris* polysaccharide CM1 [78]	LDLR(+/−) hamster	100 mg/kg/day	13.5	0.8 g/day
*C. militaris* fruiting-body powder [79]	Mouse, HFD	250 mg/kg/day	20.3	1.2 g/day
*C. guangdongensis* multi-component formula [80]	Mouse, HFD	1.5 g/kg/day	122	7.3 g/day
*P. ferulae* water extract [81]	Mouse, HFD	100 mg/kg/day	8.1	0.5 g/day
*P. ostreatus* insoluble dietary fiber [82]	Rat, HFD	1–2 g/kg/day	162–324	9.7–19.5 g/day
Mineral-enriched *P. citrinopileatus* and *H. erinaceus* mycelial tablets [83]	Mouse, STZ	6 g/kg/day	486	29 g/day
*P. abieticola* polysaccharides PAPS1 and PAPS2 [84,85]	Mouse, HFD; ApoE−/− mouse	100 mg/kg/day	8.1	0.5 g/day
*T. versicolor* heteropolysaccharides [86]	Rat, MetS model	100–300 mg/kg/day	16.2–48.6	1.0–2.9 g/day
*A. heimuer* preparation [87]	ApoE−/− mouse	500 mg/kg/day	40.5	2.4 g/day

HED values are descriptive dose-scale estimates and do not establish equivalent systemic exposure, bioavailability, safety or efficacy in humans. HED (mg/kg) = animal dose (mg/kg) × (animal Km/human Km), with Km values of 3 for mice, 5 for hamsters, 6 for rats and 37 for adults. *P. ostreatus* fruiting bodies given as 2.5–10% of the diet [88] are not included because a dose per body weight was not reported. Abbreviations: ApoE−/−, apolipoprotein E-deficient; FYGL, Fudan-Yueyang *G. lucidum* proteoglycan; GMD, ganomycin I-based meroterpene derivative; HFD, high-fat diet; LDLR, low-density lipoprotein receptor; MetS, metabolic syndrome; STZ, streptozotocin.

Taken together, preclinical findings for Cordyceps and Ophiocordyceps preparations suggest effects on glucose uptake, glycogen synthesis, the activity of carbohydrate-digesting enzymes and insulin sensitivity. Clinically, a combination of C. sinensis with G. lucidum was tested in the MetS trial described above and did not improve glycemic outcomes [58].

#### 4.1.3. Other Mushrooms (*Pleurotus ostreatus*, *Pleurotus citrinopileatus*, *Lentinula edodes*, *Hericium erinaceus*)

*Pleurotus ostreatus*, commonly known as oyster mushroom, contains bioactive compounds with potential health benefits, including antioxidant and antidiabetic activity. A methanolic extract of *P. ostreatus* inhibited α-amylase and α-glucosidase in vitro, and molecular docking suggested that selected constituents, mainly rutin and naringin, interact with amino acid residues relevant to the activity of these enzymes (Supplementary Table S1) [89]. However, inhibition was weak, reaching approximately 10% for α-amylase and 43% for α-glucosidase at the highest concentration tested (160 mg/mL), and the reported IC_50_ values (1074 and 247 mg/mL) were extrapolated beyond the tested range. These data therefore indicate, at most, a weak mechanism of uncertain physiological relevance and do not establish efficacy in vivo. In humans, small clinical trials of oyster mushroom intake, mostly in people with type 2 diabetes, reported reductions in fasting or postprandial glucose, although a systematic review rated the certainty of this evidence as low [17].

In mice with streptozotocin (STZ)-induced diabetes, buccal tablets prepared from *P. citrinopileatus* and *H. erinaceus* mycelia enriched with chromium, zinc and germanium reduced blood glucose by 29.1% relative to baseline after three weeks at the high dose (6 g/kg) and attenuated dyslipidemia and oxidative stress [83]. The relevance of this finding to MetS is limited, as STZ induces β-cell destruction and insulin deficiency rather than insulin resistance, the mineral enrichment prevents attribution of the effect to the mushroom material itself, and the effective dose corresponds to a human-equivalent dose of about 29 g/day (Table 2).

*Lentinula edodes*, known as shiitake, is an edible mushroom widely used in Asian cuisine and traditional medicine. In L6 myotubes, extracts of *L. edodes* fruiting bodies (250–1000 μg/mL) increased glucose uptake, GLUT4 expression and translocation and AMPK phosphorylation [90]. As these effects were observed in a single in vitro model, at concentrations whose physiological attainability is unknown, they constitute a mechanistic hypothesis rather than evidence of improved insulin sensitivity or body weight control. Indeed, in a randomized, double-blind trial in adults with untreated mild hypercholesterolemia, a β-glucan-enriched *L. edodes* extract (10.4 g/day providing 3.5 g of β-glucans for eight weeks) did not change fasting glucose, insulin or HbA1c compared with placebo [68].

#### 4.1.4. Summary

The available evidence suggests that selected edible and medicinal mushrooms, particularly *Ganoderma lucidum* and *Cordyceps militaris*, may influence carbohydrate metabolism by improving glucose uptake, insulin sensitivity and glycogen synthesis, and by inhibiting carbohydrate-digesting enzymes. However, these effects are supported mainly by in vitro and animal studies, often conducted at high extract concentrations or in models, such as STZ-induced diabetes, that do not reproduce insulin-resistant MetS. In humans, *G. lucidum* did not improve glycemic control in the only trial in participants with diagnosed MetS [58], meta-analytic evidence shows no effect on glycemic outcomes [19], an *L. edodes* extract did not affect glycemic markers [68], and evidence for *P. ostreatus* is of low certainty [17].

### 4.2. Lipid Profile

Disorders of lipid metabolism, characterized mainly by elevated triglyceride concentrations and reduced HDL cholesterol levels, are key components of MetS [5]. Under the harmonized definition, elevated triglycerides and reduced HDL-C constitute two separate diagnostic criteria, whereas total and LDL cholesterol, although clinically important, are not part of the definition [5]. Dyslipidemia contributes to atherosclerotic plaque formation and vascular dysfunction, and may also promote progressive cardiac dysfunction through lipid deposition in cardiomyocytes and disruption of mitochondrial metabolism [91,92].

The combination of abnormalities characteristic of MetS increases the risk of serious cardiometabolic complications, including myocardial infarction, stroke, cardiovascular disease, chronic kidney disease and type 2 diabetes [93]. Insulin resistance is one of the main mechanisms linking disturbances in glucose and lipid metabolism. Reduced insulin sensitivity may increase free fatty acid flux to the liver, enhance VLDL production and promote hypertriglyceridemia and atherogenic dyslipidemia. Dysregulated adipokine secretion from adipose tissue may further exacerbate inflammation, lipid abnormalities and metabolic dysfunction [94].

Lipid abnormalities are common among individuals with MetS, although their prevalence varies between populations and according to diagnostic criteria. Low HDL-C has been reported in 26.9–41.2% of individuals with MetS in some analyses, followed by elevated triglyceride concentrations, observed in 8.6–15.5% of participants [95]. Other studies identified low HDL-C as the most common lipid abnormality, affecting approximately 78–81% of participants in selected populations, while hypercholesterolemia was reported in up to 72% of patients with MetS [96,97].

#### 4.2.1. *Ganoderma* spp.

Preclinical data suggest a possible role for *Ganoderma lucidum* in the regulation of lipid metabolism. In a lithogenic diet-induced gallstone model, a *G. lucidum* polysaccharide (2% or 4% of the diet for six weeks) reduced gallstone formation, the cholesterol concentration in gallbladder bile and the cholesterol saturation index (Supplementary Table S2) [98]. These outcomes concern biliary cholesterol homeostasis and do not directly inform the serum lipid criteria of MetS. In mice fed a high-fat diet (HFD), sporoderm-broken spore powder (650 mg/kg/day for 12 weeks) lowered serum total cholesterol, triglycerides and LDL-C and increased HDL-C [75], and *G. lucidum* triterpenoids reduced serum triglycerides and total cholesterol [76] (Supplementary Table S3).

In humans, a randomized, double-blind, placebo-controlled trial in adults with borderline-high triglycerides (1.70–2.25 mmol/L, a range that meets the MetS triglyceride criterion) evaluated *G. lucidum* spore oil standardized to 33.7 g of triterpenoids per 100 g, given at 3.0 g/day (approximately 1.0 g of triterpenes) for 12 weeks [66]. Compared with placebo, the intervention lowered triglycerides, total cholesterol and LDL-C, raised HDL-C and improved liver function tests, without changes in renal markers (Table 1). Although the title describes a crossover study, the reported analyses were based on a parallel-group comparison (113 participants randomized; 110 analyzed). Trial registration was not reported, and between-group comparisons were based on end-of-treatment values and relative changes without baseline adjustment. In contrast, the trial in participants with MetS found no effect of *G. lucidum* on the lipid profile [58], and systematic reviews reported no significant effect on triglycerides [21], on HDL-C, LDL-C or total cholesterol in trials classified as conducted in MetS populations [59], or on the lipid profile overall [19,65]. The spore-oil result may therefore be specific to the triterpene-rich preparation rather than a general property of *G. lucidum* and requires replication.

#### 4.2.2. *Cordyceps* spp.

In 3T3-L1 adipocytes and in TSOD mice, a model of obese type 2 diabetes, *Cordyceps militaris* extract (0.1% of the diet for three weeks) and cordycepin inhibited preadipocyte differentiation through downregulation of C/EBPβ and PPARγ, promoted lipolysis and lowered LDL/VLDL cholesterol [99]. The polysaccharide CM1 isolated from *C. militaris* (100 mg/kg/day for five months) reduced plasma total cholesterol, triglycerides and apolipoprotein B48 and the epididymal fat index in LDLR(+/−) hamsters with a hyperlipidemia-like lipid profile, and inhibited preadipocyte differentiation in vitro (Supplementary Table S2) [78]. The proposed mechanisms include reduced hepatic triglyceride synthesis through downregulation of lipogenic genes, including FAS, ACC-1 and SCD-1, and reduced PCSK9 expression, which suggests an additional effect on cholesterol metabolism [78].

#### 4.2.3. Other Mushrooms (*Pleurotus ostreatus*, *Lentinula edodes*)

*Pleurotus ostreatus* was evaluated in a rat model of HFD-induced obesity, in which insoluble dietary fiber isolated from its fruiting bodies (1–2 g/kg/day for six weeks) reduced serum triglycerides, LDL-C and free fatty acids, an effect associated with the regulation of hepatic metabolic pathways, including PPAR signaling (Supplementary Table S2) [82]. In contrast, dried *P. ostreatus* powder given for four weeks to rats with prednisolone-induced hyperlipidemia produced only non-significant reductions in total cholesterol, triglycerides, LDL and VLDL [100], indicating that findings are not consistent across models and preparations. Evidence for *Lentinula edodes* in this area is limited to a single in vitro study in L6 cells, in which fruiting-body extracts increased AMPK phosphorylation and differentially regulated PPARγ and FASN expression [90].

In humans, oyster mushroom consumption has been evaluated in small clinical trials, mostly as a whole food in people with type 2 diabetes or hyperlipidemia, and a systematic review concluded that the evidence for a cardiometabolic benefit is of low certainty [17]. For *L. edodes*, two randomized, double-blind, placebo-controlled trials yielded inconsistent results. Bars containing shiitake consumed for 66 days by 68 adults with borderline-high lipid levels reduced triglycerides by 10% within the intervention group and improved markers of oxidative stress, although dermatitis occurred in 10% of participants [69]. In contrast, a β-glucan-enriched *L. edodes* extract providing 3.5 g of β-glucans per day for eight weeks did not change total cholesterol, LDL-C, HDL-C or triglycerides compared with placebo in 52 adults with untreated mild hypercholesterolemia [68]. Consistently, a systematic review of whole-mushroom consumption found limited evidence for lower triglycerides and no consistent effects on other lipid parameters [18].

#### 4.2.4. Summary

Current findings suggest that *Ganoderma lucidum*, *Cordyceps militaris*, *Pleurotus ostreatus* and *Lentinula edodes* may affect lipid metabolism in experimental models through mechanisms related to PPAR signaling, adipogenesis, lipolysis, hepatic lipogenesis and cholesterol regulation, although results are not consistent across models. Clinical evidence is also inconsistent. A single trial of triterpene-rich *G. lucidum* spore oil improved the lipid profile in adults with borderline-high triglycerides [66], and shiitake bars reduced triglycerides within the intervention group [69]. However, the trial in participants with MetS, systematic reviews of *G. lucidum* and a placebo-controlled trial of a *L. edodes* extract found no effect on lipids [19,58,68], and the certainty of evidence for *P. ostreatus* is low [17].

### 4.3. Body Weight and Adipose Tissue

Abdominal obesity is one of the diagnostic criteria for MetS and significantly increases the risk of cardiovascular disease, type 2 diabetes and mortality [101,102]. In the harmonized definition, this criterion is assessed by waist circumference according to population- and country-specific thresholds [5], whereas preclinical studies often report body weight and fat-pad mass, and most human studies report BMI. Changes in body weight or BMI therefore cannot be equated with improvement in the adiposity criterion of MetS. The high prevalence of obesity and its continuing increase indicate the global importance of this health problem. In some populations, the prevalence of obesity may reach 45.1%, while projections suggest that by 2030 nearly 58% of adults may have overweight or obesity [4,103].

Excess body weight is associated with several metabolic complications, including insulin resistance, which plays a key role in the pathogenesis of MetS. Excess visceral adipose tissue promotes adipocyte dysfunction, macrophage infiltration and increased production of pro-inflammatory cytokines in adipose tissue, the liver and skeletal muscle [103,104,105,106]. These changes impair insulin signaling, reduce glucose utilization in skeletal muscle and increase hepatic glucose production, thereby exacerbating metabolic dysfunction [103,107].

In metabolically healthy adipose tissue, anti-inflammatory M2 macrophages support insulin sensitivity. However, chronic nutrient excess, adipocyte hypertrophy and cellular stress promote a shift toward the pro-inflammatory M1 phenotype, contributing to chronic low-grade inflammation and obesity-related insulin resistance [104].

#### 4.3.1. *Ganoderma* spp.

Preclinical studies suggest that *Ganoderma lucidum* preparations may influence body weight and adipose tissue accumulation, although the evidence is limited to animal models (Supplementary Table S3). In Swiss mice, an ethanolic extract of *G. lucidum* reduced body weight gain at 100 and 200 mg/kg per day over 30 days. This effect was accompanied by a decrease in feed intake, whereas water intake increased with increasing dose [73], so reduced food consumption may have contributed to the observed weight change. Sang et al. showed that a polysaccharide isolated from sporoderm-broken spores of *G. lucidum* (100 or 300 mg/kg for 12 weeks) reduced diet-induced adipose tissue deposition, body weight gain, inflammation and hepatic steatosis in mice fed a high-fat diet (HFD). These effects correlated with the restoration of gut microbiota homeostasis, suggesting a microbiota-mediated mechanism that warrants testing in humans [74]. Sporoderm-broken spore powder (650 mg/kg/day by oral gavage for 12 weeks) also reduced body weight gain, liver weight gain and epididymal fat accumulation without changing food intake, and its effect on epididymal fat was comparable to that of the L-carnitine positive control [75]. Triterpenoids extracted from the fruiting body of *G. lucidum* (100 or 200 mg/kg/day for 12 weeks) reduced body weight gain, serum triglycerides and total cholesterol, epididymal fat accumulation and adipocyte size in HFD-fed mice and inhibited 3T3-L1 preadipocyte differentiation through PRKCQ-related signaling [76]. Resinacein S, a triterpenoid isolated from *Ganoderma resinaceum*, increased heat production and energy expenditure and inhibited body weight gain in mice [108]. Finally, a synthetic meroterpene derivative designed on the basis of ganomycin I (GMD), which is not a dietary constituent, was given to ApoE−/− mice after eight weeks of a high-fat diet (5 or 10 mg/kg/day for nine weeks). It reduced body weight gain, insulin resistance, hyperlipidemia and atherosclerotic lesion area, an effect attributed to an increase in the intestinal abundance of *Parabacteroides merdae*, which enhanced the catabolism of branched-chain amino acids and modulated mTORC1 signaling [77]. Across these studies, effects reported for *G. lucidum* derive from an ethanolic extract, a spore polysaccharide, spore powder and a triterpenoid fraction, which precludes attributing the anti-obesity effect to a single defined constituent.

#### 4.3.2. *Cordyceps* spp.

*Cordyceps* spp. have also been investigated for their effects on body weight and adipose tissue, predominantly in preclinical animal models (Supplementary Table S3), as is the case for *Ganoderma* spp.

Cordycepin is a major constituent of *Cordyceps militaris*. In 3T3-L1 adipocytes and TSOD mice, *C. militaris* extract and cordycepin limited the formation of new adipocytes and enhanced the breakdown of accumulated fat, partly by reducing oxidative stress and cellular senescence in adipocytes [99]. In mice fed a high-fat diet, both conventional and selenium-enriched dried fruiting bodies of *C. militaris* (250 mg/kg/day for 12 weeks, corresponding to approximately 1.2 g/day for a 60 kg adult) limited body weight gain and reduced liver weight and the mass of epididymal, perirenal and inguinal adipose tissue, with no difference between the two materials. Both preparations also lowered fasting glucose, improved glucose tolerance and reduced serum triglycerides, total cholesterol and LDL-C. However, energy intake was reduced as well, which the authors considered a possible explanation for the effect on body weight [79].

In a study by Wang et al., a multi-component lipid-lowering formula containing fruiting body powder of *Cordyceps guangdongensis* (1.5 g/kg/day for 14 weeks) reduced body weight and fat deposition in HFD-fed mice. Moreover, it improved glucose tolerance and the lipid profile and limited hepatic steatosis. Administration of this formulation increased the production of short-chain fatty acids (SCFAs) and regulated the expression of genes associated with lipid metabolism. Changes in the composition of the gut microbiota were also observed, including an increase in the abundance of bacteria from the genera *Bacteroides* (*B. acidifaciens*) and *Bifidobacterium (B. pseudolongum*), with a concomitant reduction in the proportions of *Dubosiella, Ligilactobacillus* and *Romboutsia* [80]. Because the formula contained other components, the contribution of *C. guangdongensis* itself cannot be isolated.

#### 4.3.3. Other Mushrooms (*Pleurotus ostreatus*, *Pleurotus ferulae*)

A hot-water extract of *Pleurotus ferulae* (PWE, 100 mg/kg/day in drinking water for eight weeks) reduced body weight gain, fat mass, adipocyte size and serum total cholesterol and LDL-C in HFD-fed mice, and at 100–200 μg/mL it inhibited lipid accumulation and the expression of PPARγ, C/EBPα and lipogenic genes in 3T3-L1 adipocytes [81]. The ethanolic extract (PEE) was assessed only in a preliminary 3T3-L1 screen, in which it inhibited lipid accumulation less effectively than PWE, and it was not tested in vivo. The authors attributed the activity of PWE to polysaccharides, which were more abundant in PWE than in PEE, whereas phenolics and flavonoids were not detected in either extract. The in vivo outcomes were, however, reported for three animals per group, which limits their statistical robustness [81].

In mice with obesity established by six weeks of HFD feeding, dried *P. ostreatus* fruiting bodies incorporated into the HFD at 2.5%, 5% and 10% (*w*/*w*) for a further six weeks inhibited body weight gain and white adipose tissue deposition, with stronger effects at higher doses, and maintained glucose homeostasis. The intervention also shifted the composition and predicted functional pathways of the gut microbiota towards those of lean mice, suggesting that its activity is partly dependent on microbiota modulation [88].

#### 4.3.4. Summary

Preclinical studies suggest that *Ganoderma lucidum, Cordyceps militaris, Cordyceps guangdongensis*, *Pleurotus ferulae* and *Pleurotus ostreatus* may reduce body weight gain, adipose tissue accumulation and obesity-related metabolic disturbances. The reported effects may involve modulation of lipid metabolism, inflammatory pathways, gut microbiota composition and short-chain fatty acid production. However, most of these studies used preventive designs in HFD-fed rodents and preparations ranging from whole fruiting bodies to isolated or synthetic compounds. Human data are scarce and do not indicate an effect on central adiposity. A small pooled reduction in BMI (−0.43 kg/m^2^) with *G. lucidum* was not accompanied by changes in waist circumference or body fat [19], the trial in participants with MetS found no effect on waist circumference or BMI [58], and a *L. edodes* extract did not change body weight, BMI or waist circumference compared with placebo [68]. The relevance of the preclinical findings for the adiposity criterion of MetS therefore remains to be established.

### 4.4. Blood Pressure

Elevated blood pressure is a common component of MetS and is closely linked to the overall cardiometabolic risk profile [109]. For MetS diagnosis, elevated blood pressure is defined as systolic blood pressure ≥ 130 mmHg and/or diastolic blood pressure ≥ 85 mmHg, or the use of antihypertensive medication [5]. This threshold should be distinguished from the definition of hypertension in the 2024 European Society of Cardiology guidelines, which uses confirmed office blood pressure of ≥140/90 mmHg [110]. The prevalence of MetS increases with hypertension severity, and approximately 80% of patients diagnosed with MetS also have hypertension [109]. Importantly, MetS is associated with an approximately twofold higher risk of cardiovascular events and about a 50% increase in all-cause mortality compared with individuals without this syndrome [111].

The development of hypertension in MetS is multifactorial and involves insulin resistance, excess body weight, increased sympathetic nervous system activity and sodium retention related to enhanced renal tubular reabsorption [109]. Chronic blood pressure elevation contributes to cardiovascular remodeling and is associated with RAAS disturbances, ischemic complications and increased risk of myocardial infarction [112].

Angiotensin-converting enzyme (ACE), which converts angiotensin I into angiotensin II, is important in the regulation of blood pressure. Angiotensin II promotes vasoconstriction and increases blood pressure, while other enzymes, including arginase, cholinesterase and adenosine deaminase, may also influence vascular and blood pressure-related mechanisms [113].

#### 4.4.1. Other Mushrooms (*Pleurotus ostreatus*, *Lentinus subnudus*, *Trametes versicolor*)

Aqueous extracts of *Pleurotus ostreatus* and *Lentinus subnudus* were evaluated for their effects on enzymes associated with blood pressure regulation (Supplementary Table S4). In lung and heart homogenates from Wistar rats, both extracts (1–25 μg/mL) significantly and concentration-dependently reduced the activity of angiotensin-converting enzyme and arginase, with a stronger effect of *P. ostreatus* on both enzymes, whereas *L. subnudus* showed greater inhibition of cholinesterase [113]. Enzyme inhibition in tissue homogenates indicates a potential mechanism but does not demonstrate an effect on blood pressure in vivo.

A preclinical study of *Trametes versicolor* heteropolysaccharides (TVH) was conducted in Wistar rats in which MetS was induced by a four-week high-fat diet followed by a single low dose of streptozotocin and confirmed by hyperglycemia, hyperinsulinemia, dyslipidemia and elevated blood pressure. TVH, a commercial preparation characterized by FT-IR spectroscopy, GC-MS monosaccharide analysis and HPLC molecular weight determination, was given orally at 100, 200 or 300 mg/kg for four weeks. All three doses lowered systolic and diastolic blood pressure without a dose-dependent effect, whereas improvements in glucose tolerance and insulin levels were significant only at the highest dose, and effects on lipids, redox status and cardiac function were most pronounced at the two higher doses [86]. This study is notable for combining a chemically characterized preparation with a model in which several MetS components were present simultaneously, although the use of streptozotocin, even at a low dose, adds an element of β-cell injury that is not typical of human MetS.

#### 4.4.2. Summary

Evidence regarding the effects of mushrooms on blood pressure regulation remains limited. In experimental studies, *P. ostreatus* and *L. subnudus* extracts inhibited ACE and related enzymes in tissue homogenates, and *T. versicolor* heteropolysaccharides lowered blood pressure in a rat model of MetS. In humans, the trial in participants with MetS [58] and systematic reviews [19,21] found no significant effect of *G. lucidum* on blood pressure, and a *L. edodes* extract did not affect blood pressure compared with placebo [68]. The antihypertensive relevance of the preclinical findings therefore remains uncertain.

### 4.5. Inflammatory Markers and Chronic Inflammation

The pathogenesis of MetS is multifactorial, with chronic low-grade inflammation closely linked to obesity, insulin resistance and oxidative stress [114]. Inflammatory and oxidative stress markers are not diagnostic criteria for MetS, and changes in these markers are therefore discussed here as mechanistic or related outcomes rather than diagnostic endpoints. Excess adipose tissue promotes increased secretion of inflammatory mediators, including IL-6, IL-1, CRP and TNF-α, while insulin resistance and oxidative stress may further activate inflammatory cascades, endothelial dysfunction and atherosclerotic processes [1,115]. In a study involving 179 women aged 43–73 years, higher inflammatory biomarker levels were observed in MetS, including increased IL-6 in women with insulin resistance and hypertriglyceridemia and a positive correlation between TNF-α and abdominal obesity [116]. Persistent systemic inflammation can adversely affect adipose tissue, pancreas, liver, skeletal muscle, brain and heart and is associated with cardiovascular disease, hypertension and insulin resistance [117,118].

#### 4.5.1. *Ganoderma* spp.

Preclinical studies suggest that *Ganoderma lucidum* preparations may modulate inflammatory and oxidative stress-related markers, although the available data derive mainly from in vitro studies and animal models (Supplementary Table S5). Isolated *G. lucidum* triterpenoids, namely lucidone D, ganoderterpene A and ganoderic acid A, reduced the production of TNF-α, IL-1β, IL-6 and nitric oxide and the expression of inducible nitric oxide synthase (iNOS) and cyclooxygenase-2 (COX-2) [119,120,121]. However, these effects were obtained in LPS-stimulated macrophages and microglia and in IL-1β-treated nucleus pulposus cells, that is, in models unrelated to metabolic disease, so they indicate general anti-inflammatory activity rather than effects on metabolic inflammation. Data closer to the cardiometabolic context come from a carotid artery ligation model of disturbed flow-induced atherogenesis, in which oral *G. lucidum* (300 mg/kg/day) prevented neointimal thickening and a subcutaneously administered triterpenoid extract abolished neointima formation, whereas in human umbilical vein endothelial cells exposed to disturbed flow the triterpenoids reduced the expression of vascular cell adhesion molecule-1 (VCAM-1), TNF-α and IL-6 [122]. In HFD-fed mice, sporoderm-broken spore powder (650 mg/kg/day for 12 weeks) decreased the expression of TNF-α, IL-6 and IL-1β [75].

Human data on inflammatory and oxidative stress outcomes come from small trials of different *G. lucidum* preparations conducted in adults with cardiometabolic syndrome defined by NCEP-ATP III criteria [60], individuals with obesity defined by BMI [62], patients with paroxysmal atrial fibrillation [63] and older women without neurodegenerative diseases [64] (Table 1). Their primary outcomes concerned inflammatory, oxidative stress, immune or renal markers rather than MetS diagnostic components, sample sizes ranged from 38 to 68 participants, and all four trials were rated as being of poor methodological quality in the meta-analysis by Jafari et al. The pooled analysis of inflammatory markers in that meta-analysis showed no significant effect [19].

In older women, supplementation with a *G. lucidum* dry extract was associated with lower percentages of Th17 cells and higher expression of regulatory T-cell-associated genes, including FOXP3, IL-10 and TGF-β, compared with placebo [64]. In patients with paroxysmal atrial fibrillation, GLPP reduced MDA, hs-CRP, TNF-α, IL-1β and IL-6 more than placebo [63]. In contrast, in individuals with obesity, a mycelial β-glucan produced no significant between-group differences in TNF-α, MDA, nitric oxide, urea or creatinine, as these markers changed similarly in both groups [62]. In adults with cardiometabolic syndrome, GLPP was associated with a decrease in MDA and an increase in SOD within the treatment group, whereas TNF-α and hs-CRP declined in both groups, and only within-group comparisons were reported [60].

#### 4.5.2. *Cordyceps* and *Ophiocordyceps* spp.

Two in vitro studies examined specific *Cordyceps sinensis* preparations, including an ethanolic extract (CSE) [123] and EPS-LM, an exopolysaccharide obtained from mycelial fermentation of the *C. sinensis* Cs-HK1 strain [124]. These preparations reduced selected inflammatory and oxidative stress-related markers and modulated TLR4/MyD88/NF-κB- and Nrf2/HO-1-related signaling pathways (Supplementary Table S5) [123,124]. No clinical studies were identified for these specific *Cordyceps sinensis*-related preparations. The LPS challenge in piglets, however, models acute intestinal inflammation rather than metabolic inflammation.

*Cordyceps militaris* has also been investigated for its effects on inflammation and oxidative stress. In LPS-challenged piglets, supplementation with *C. militaris* extract or cordycepin altered the ileal cytokine profile, with lower IL-6, IL-8, TNF-α and IL-10 levels than in the LPS group. Cordycepin also counteracted the LPS-induced reduction in serum IL-10 but increased serum TNF-α relative to the LPS group (Supplementary Table S5) [125]. Both interventions modulated the intestinal microbiota and increased the abundance of selected SCFA-producing bacteria, whereas a significant reversal of the LPS-associated reduction in ileal butyrate concentration was observed only with cordycepin [125]. Clinical evidence concerning *Cordyceps*-related preparations comprises the combination of *C. sinensis* with *G. lucidum* tested in the MetS trial, which showed no metabolic benefit [58], and a single trial of CBG-CS-2, a mycelial culture extract of *Paecilomyces hepiali*, in healthy adults, in which supplementation was associated with increased NK-cell activity, whereas no significant between-group differences were observed in the analyzed cytokines (Table 1) [67].

#### 4.5.3. Other Mushrooms (*Pleurotus abieticola*, *Auricularia heimuer*)

Preclinical studies have also reported modulation of inflammatory and oxidative stress-related responses by *Pleurotus abieticola* and *Auricularia heimuer*, in addition to *Ganoderma lucidum* and the *Cordyceps*- and *Ophiocordyceps*-related preparations described above (Supplementary Table S5). *Pleurotus abieticola* inhibited signaling pathways, including NF-κB/signal transducer and activator of transcription 3 (STAT3) and TLR4/NF-κB [84,85]. Reductions in hepatic ROS levels and pro-inflammatory cytokines, including TNF-α, IL-1β, IL-6, IL-17A, IL-22 and IL-23, were also reported [84]. In addition, administration of *P. abieticola* to mice fed a high-fat diet led to increased levels of IL-10, a pleiotropic cytokine with important immunoregulatory functions, as well as Nrf2 and the antioxidant enzymes SOD and glutathione peroxidase (GSH-Px) [84,85]. In turn, *Auricularia heimuer* reduced ROS in the vascular endothelium and increased the activity of Nrf2, HO-1, SOD-1 and IL-10 [87]. Importantly, this mushroom was also reported to reduce the phosphorylation levels of NF-κB, IL-6, leukotriene B4 receptor 2 (LTB4R2), inhibitor of kappa B alpha (IκBα) and IκB kinase complex α/β (IKKα/β) [87].

#### 4.5.4. Summary

Most evidence on anti-inflammatory and antioxidant effects concerns *Ganoderma lucidum*, *Cordyceps militaris*, and specific preparations reported under the name *Cordyceps sinensis*, whereas data for *Pleurotus abieticola* and *Auricularia heimuer* remain predominantly preclinical. The reported effects include modulation of cytokine production, oxidative stress markers and NF-κB/Nrf2-related pathways, although many were obtained in models of acute inflammation unrelated to metabolic disease. In humans, inflammatory and oxidative stress outcomes have been assessed only in small trials of heterogeneous populations and preparations, and the pooled analysis showed no significant effect on inflammatory markers [19]. The heterogeneity of mushroom preparations, doses and study populations further limits direct comparison of results.

### 4.6. Overview of the Evidence

**Figure 1:**
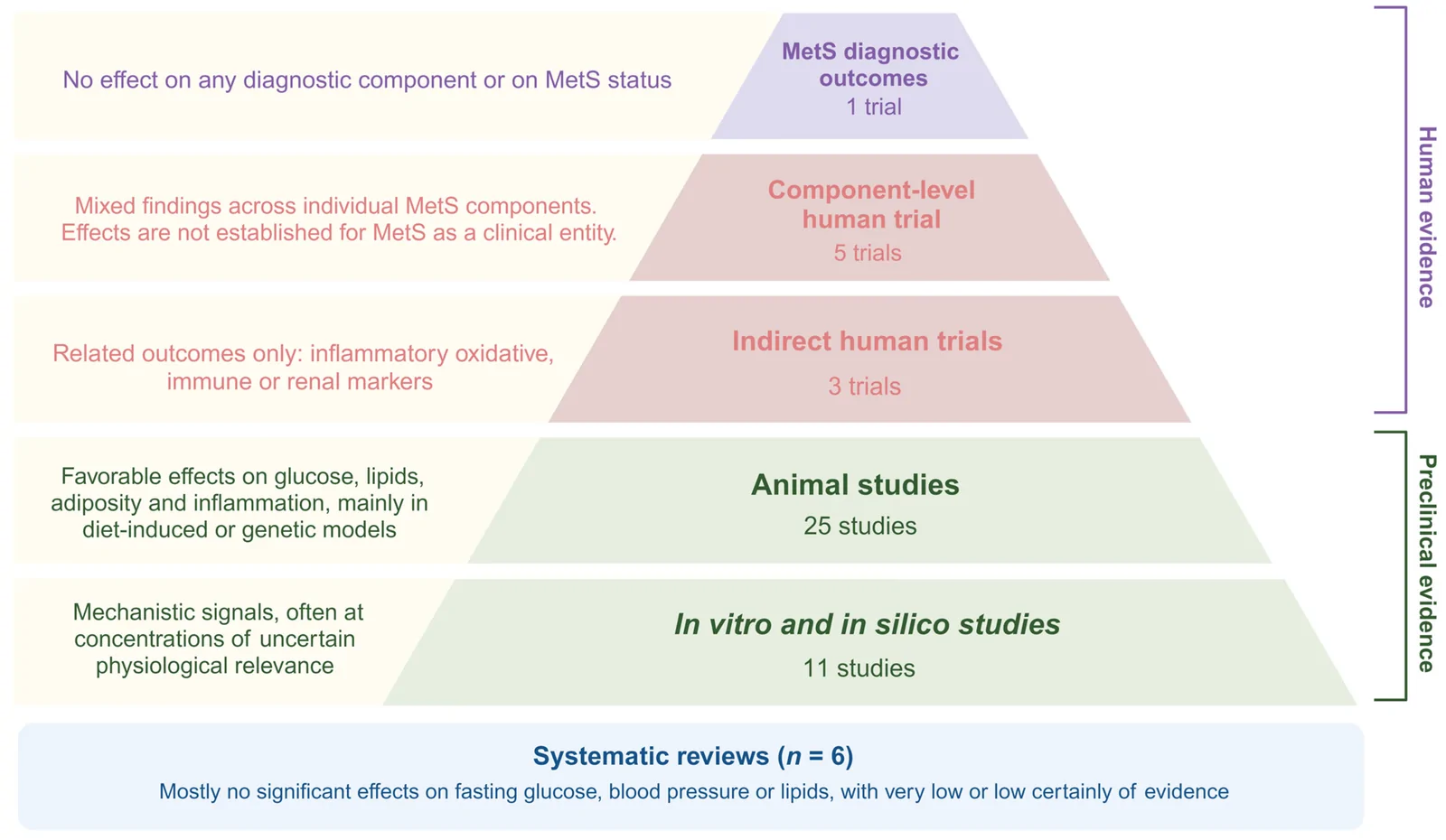
Distribution of the evidence on edible and medicinal mushrooms in relation to metabolic syndrome (MetS) by level of evidence. Human trials are classified by their directness to MetS as defined in Section 2.3. Numbers refer to the studies summarized in Table 1 and Supplementary Tables S1–S5. Studies combining animal and in vitro experiments were counted in the animal tier. Created with BioRender.com.

Table 3 maps the available evidence by mushroom species, preparation type, outcome, and level of investigation, while Figure 1 summarizes the distribution and directness of preclinical and clinical evidence relevant to MetS. Preclinical findings concern glucose and lipid metabolism, adiposity, vascular-related enzyme activity and inflammatory pathways; however, direct assessments of blood pressure remain scarce. In studies involving mushroom-derived preparations, the available vascular evidence includes enzyme inhibition in tissue homogenates [113] and blood pressure measurements in a rat model of MetS [86]. Human evidence is concentrated on *Ganoderma lucidum*. The only randomized trial assessing diagnostic components in participants with established MetS reported no improvement in metabolic outcomes [58], consistent with the predominantly null findings of systematic reviews concerning glycemic parameters, blood pressure, and lipids [19,21,59,65]. Evidence for edible species such as *Pleurotus ostreatus* and *Lentinula edodes* is limited and heterogeneous [17,68,69]. Other species reviewed here have not been evaluated in the identified clinical trials assessing MetS diagnostic components.

## 5. Discussion

**Figure 2:**
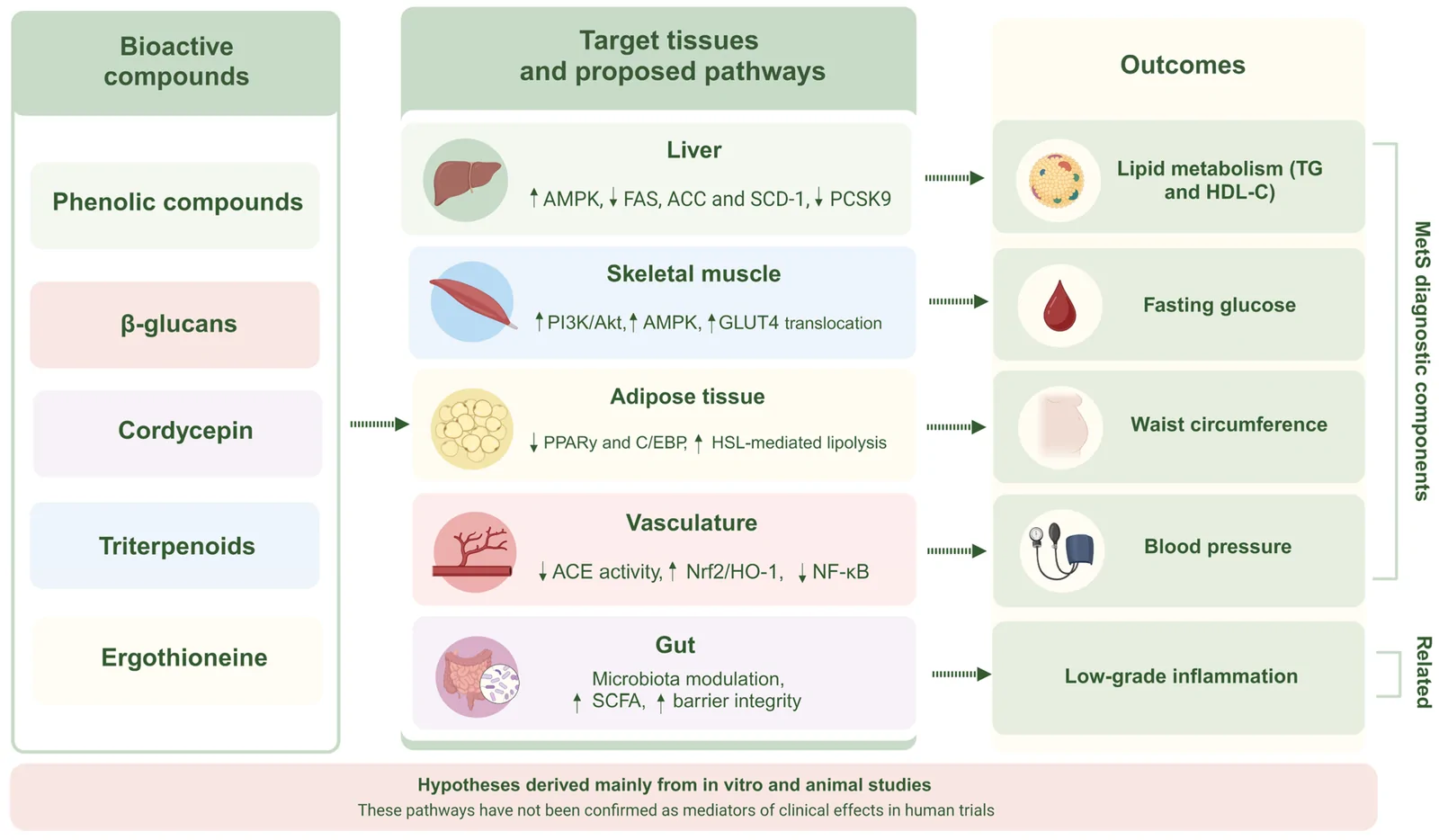
Proposed mechanisms linking mushroom-derived compounds to the components of metabolic syndrome (MetS), based predominantly on in vitro and animal studies. Arrows indicate the outcome to which each pathway has been most directly linked in experimental studies, although most pathways may influence several outcomes. Created with BioRender.com.

Edible and medicinal mushrooms such as *Ganoderma lucidum*, *Ophiocordyceps sinensis*, *Cordyceps militaris*, *Pleurotus abieticola*, *Pleurotus ostreatus*, *Lentinula edodes* and *Auricularia heimuer* modulated key biological processes involved in MetS in experimental models. The proposed mechanisms are diverse and involve glucose and lipid homeostasis, broader metabolic processes and immune responses [126]. Before examining these mechanisms as hypotheses that may explain preclinical findings (Section 5.2, Section 5.3, Section 5.4 and Section 5.5), the human evidence is appraised (Section 5.1), and the discussion then turns to the translational validity of the preclinical data (Section 5.6), the heterogeneity of the preparations studied (Section 5.7) and the limitations of this review (Section 5.8). The proposed mechanisms are summarized in Figure 2.

ACC, acetyl-CoA carboxylase; ACE, angiotensin-converting enzyme; AMPK, AMP-activated protein kinase; C/EBP, CCAAT/enhancer-binding protein; FAS, fatty acid synthase; GLUT4, glucose transporter 4; HDL-C, high-density lipoprotein cholesterol; HO-1, heme oxygenase-1; HSL, hormone-sensitive lipase; NF-κB, nuclear factor κB; Nrf2, nuclear factor erythroid 2-related factor 2; PCSK9, proprotein convertase subtilisin/kexin type 9; PI3K/Akt, phosphoinositide 3-kinase/protein kinase B; PPARγ, peroxisome proliferator-activated receptor γ; SCD-1, stearoyl-CoA desaturase 1; SCFA, short-chain fatty acids.

### 5.1. Critical Appraisal of the Human Evidence

Human evidence relevant to MetS can be organized according to its directness (Table 1). Direct evidence is limited to one randomized, double-blind, placebo-controlled trial in 84 adults with type 2 diabetes and MetS defined by NCEP-ATP III criteria [58]. Neither *G. lucidum* (3 g/day) nor its combination with *C. sinensis* affected HbA1c, fasting glucose, blood pressure, triglycerides, HDL-C, waist circumference, BMI or C-reactive protein over 16 weeks, and the number of participants meeting MetS criteria did not decrease. The trial was prospectively registered and analyzed on an intention-to-treat basis with adjustment for baseline values, and the observed effects did not approach the predefined smallest clinically worthwhile differences, although the planned sample size was not reached [58]. A second trial enrolled participants with MetS defined by the same criteria, but it assessed only inflammatory, oxidative stress and renal markers and reported within-group comparisons [60].

Component-level evidence comes from systematic reviews and individual trials. A Cochrane review found no effect of *G. lucidum* on HbA1c, blood pressure, triglycerides or cholesterol, although all analyzed trials were conducted in people with type 2 diabetes [21]. A later review found no differences in HDL-C, LDL-C, total cholesterol or fasting glucose in trials it classified as conducted in MetS populations [59], and a systematic review of seven trials found no effect on triglycerides, HDL-C or LDL-C [65]. The most comprehensive meta-analysis to date pooled 17 randomized trials with 971 participants, six of which tested multi-ingredient products in which *G. lucidum* was combined with other herbs [19]. It found no significant effect on fasting glucose, blood pressure, lipid profile, waist circumference (weighted mean difference [WMD] −0.45 cm, 95% CI −1.02 to 0.12) or body fat, together with small reductions in BMI (WMD −0.43 kg/m^2^, 95% CI −0.77 to −0.10) and serum creatinine (WMD −0.14 mg/dL, 95% CI −0.27 to −0.02), and it rated the certainty of evidence as very low for all outcomes [19]. A BMI reduction in this magnitude, unaccompanied by a change in waist circumference, does not indicate an improvement in the central adiposity criterion of MetS. Serum creatinine is not a diagnostic criterion for MetS. It is included in Table 1 because it was reported by the meta-analysis and because chronic kidney disease is a recognized complication of MetS [93]. However, the pooled reduction in serum creatinine was not robust in sensitivity analyses, as its statistical significance was lost after the separate exclusion of trials by Wardhani et al. [60], Chen et al. [61], or Sargowo et al. [62]. The clinical relevance of this small decrease in populations with largely normal renal function remains uncertain. Furthermore, the subsequent spore-oil trial found no change in renal markers [66].

Overlap between primary and secondary evidence was assessed explicitly (Table 1). The MetS trial [58] was included in four of the systematic reviews [19,21,59,65], and four other primary *G. lucidum* trials [60,62,63,64] were pooled in the meta-analysis by Jafari et al. [19], which covered the literature up to August 2024. The spore-oil trial [66] was published afterwards, and the *P. hepiali* [67] and *L. edodes* trials [68,69] concerned other species. The overlapping trials were not re-analyzed, and their individual results are presented only to describe populations, preparations and outcome domains not captured by pooled estimates.

The methodological quality of the trials varied substantially (Table 1). Apart from the MetS trial, most were small (38–113 participants) and short (8–16 weeks), only a subset were prospectively registered, and several analyzed participants per protocol, reported only within-group changes, or did not describe randomization or blinding [60,62,63,64,66,67]. Most trials were rated as being of poor quality in the meta-analysis by Jafari et al. [19]. Within-group changes alone do not establish treatment efficacy without an appropriate between-group comparison. The preparations ranged from triterpene-standardized spore oil to polysaccharide peptides, a mycelial β-glucan and dry extracts, and the extent of their chemical characterization varied from a declared content of β-glucans or triterpenoids to a full proximate composition [60,62,63,64,66,67,68].

Taken together, the human evidence does not show that any mushroom preparation improves MetS as a clinical entity. Positive findings are confined to single, unreplicated trials of specific preparations and to related markers, whereas the trial with the most rigorous design and the most directly relevant population reported no significant effects.

### 5.2. Regulation of Energy Metabolism

Disturbances in energy metabolism are closely associated with insulin resistance, dyslipidemia and excess adipose tissue accumulation in MetS. Under conditions of chronic energy excess, lipids may accumulate in the liver, skeletal muscle and pancreas, contributing to lipotoxicity, mitochondrial dysfunction and impaired metabolic flexibility [127]. These processes may further intensify oxidative stress and insulin resistance, creating a self-reinforcing cycle of metabolic dysfunction.

AMPK is a key regulator of cellular energy homeostasis and is involved in glucose utilization, fatty acid oxidation, gluconeogenesis and lipid synthesis [128,129]. By modulating these processes, AMPK may influence several abnormalities characteristic of MetS, including impaired insulin sensitivity, excessive hepatic lipid accumulation, oxidative stress and chronic inflammation. Bioactive compounds present in edible and medicinal mushrooms may affect AMPK-related pathways, which could partly explain the beneficial metabolic effects observed in experimental studies [130].

Mitochondrial dysfunction is another proposed link between energy imbalance and MetS. Increased glucose availability, lipid accumulation and oxidative stress may impair mitochondrial function and contribute to pancreatic β-cell dysfunction, altered fatty acid metabolism and reduced insulin sensitivity [131,132,133,134]. Mushroom-derived compounds, including β-glucans, triterpenes, eritadenine and phenolic compounds, have been suggested to support metabolic balance by reducing ROS accumulation, oxidative stress and endoplasmic reticulum stress [38,135]. In addition, inhibition of lipogenesis and stimulation of β-oxidation may be associated with adiponectin- and AMPK-related regulation of enzymes involved in lipid metabolism, including ACC-1, FAS and SREBP-1c, as well as PGC-1α, which is involved in mitochondrial biogenesis [136].

Nevertheless, direct evidence that edible or medicinal mushroom supplementation improves energy metabolism or mitochondrial function in MetS remains limited. Most findings are derived from experimental models, and further clinical studies are needed to determine whether these mechanisms are relevant in humans.

### 5.3. Modulation of Oxidative Stress and Chronic Inflammation

Oxidative stress and chronic low-grade inflammation are central mechanisms linking obesity, insulin resistance, endothelial dysfunction and cardiometabolic complications in MetS [137]. In patients with MetS, oxidative stress has been associated with mitochondrial dysfunction, protein damage, lipid peroxidation and impaired antioxidant defense systems [138]. Bioactive compounds present in edible and medicinal mushrooms, including polysaccharides, peptides, terpenoids and polyphenols, may modulate these processes by reducing ROS accumulation, limiting lipid peroxidation and supporting endogenous antioxidant defense mechanisms [139,140]. The Nrf2 pathway is an important component of the cellular antioxidant response. Nrf2 regulates the expression of antioxidant enzymes, including SOD and GSH-Px, which participate in ROS neutralization and protection against lipid peroxidation products such as MDA [85,141]. Activation of Nrf2-related pathways, increased antioxidant enzyme activity and reduced inflammatory responses have been reported in experimental models for several mushroom-derived preparations, including polysaccharides from *P. abieticola* and *A. heimuer* [85,87] and steroids from the edible mushroom *Hypholoma lateritium* [142].

Chronic inflammation in MetS is also associated with NF-κB signaling. This pathway is activated by inflammatory stimuli, including TNF-α, IL-1β and metabolic stress, and contributes to the expression of pro-inflammatory mediators [143,144]. Pro-inflammatory mediators such as IL-6 may further activate the IKK/NF-κB axis, whereas IL-10 participates in the inhibition of excessive inflammatory responses [145,146]. β-glucans and other mushroom-derived compounds may attenuate inflammatory signaling by modulating NF-κB-related pathways and reducing the expression or activity of inflammatory mediators, including TNF-α, IL-1β, IL-6, iNOS and COX-2 [119,120,121].

Mushroom-derived compounds may also influence immune regulation, including pathways related to Th17 and regulatory T-cell responses, which are relevant to chronic inflammation and immunometabolic regulation [64]. However, most evidence remains preclinical. Human studies involve heterogeneous preparations, doses, populations and endpoints, and pooled clinical analyses have shown no significant effects on inflammatory markers [19].

### 5.4. Regulation of Insulin Signaling and Glucose Metabolism

Disturbances in insulin signaling and glucose metabolism are closely linked to MetS, although it remains unclear whether impaired carbohydrate metabolism is a primary driver or a consequence of broader metabolic and inflammatory alterations [53,147]. Under physiological conditions, insulin binding to its receptor activates intracellular signaling pathways, including PI3K/Akt and ERK, which regulate glucose uptake, glycogen synthesis, lipogenesis and gluconeogenesis [148]. In MetS, insulin resistance may further interact with chronic inflammation, altered adipokine secretion, mitochondrial dysfunction and oxidative stress, thereby exacerbating disturbances in glucose homeostasis [149,150].

Preclinical studies suggest that edible and medicinal mushrooms may influence insulin-related pathways. Increased GLUT4 mRNA expression has been reported in skeletal muscle and adipose tissue in experimental models, suggesting a possible role in glucose transport into insulin-sensitive tissues [151]. Mushroom polysaccharides may also affect insulin signaling through modulation of PI3K/Akt, JNK and MAPK pathways [152]. These mechanisms may help explain improvements in glucose uptake and insulin sensitivity observed in selected in vitro and animal studies.

In addition to intracellular insulin signaling, edible mushrooms may influence carbohydrate metabolism by inhibiting α-amylase and α-glucosidase, enzymes involved in carbohydrate digestion and monosaccharide release [153,154]. Such inhibition may delay glucose absorption and reduce postprandial glycemic excursions. In vitro studies indicate that this activity may be related to the diverse composition of mushroom-derived compounds, including organic acids, phenolic acids and fatty acids [155]. However, clinically meaningful effects on glucose metabolism have yet to be established in human studies.

### 5.5. The Gut-Metabolism Axis

Polysaccharides derived from edible and medicinal mushrooms may influence metabolic health through effects on the gut microbiota. Their effects have been linked to increased SCFA production, support of intestinal barrier integrity and regulation of lipid metabolism [156]. Dietary fiber from edible mushrooms, including *Lentinula edodes* and *Hericium erinaceus*, has also been investigated as a potential prebiotic component capable of modifying gut microbiota composition [157].

Mushroom-derived compounds, particularly β-glucans and triterpenoids, may alter microbial composition by increasing the abundance of potentially beneficial bacteria, including *Bifidobacterium* and *Lactobacillus*, while reducing the abundance of *Enterobacteriaceae* [158]. Increased SCFA production may support intestinal barrier function, attenuate inflammation, promote regulatory T-cell differentiation and inhibit TLR4/NF-κB-related signaling. In mice fed a Western diet, cordycepin attenuated metabolic inflammation, an effect attributed to improved intestinal barrier integrity and modulation of the gut microbiota [159].

Preclinical studies also suggest that *Ganoderma lucidum* polysaccharides may increase SCFA production by stimulating SCFA-producing bacteria, including *Ruminococcus*_1 and *Paraprevotella* [160]. Edible mushroom polysaccharides have also been associated with changes in the relative abundance of *Bacteroidetes* and *Firmicutes*, microbial groups often discussed in the context of obesity and metabolic dysregulation [160,161]. Together, these findings suggest that mushroom-derived polysaccharides may influence the gut-metabolism axis through changes in microbial composition, SCFA production and inflammatory signaling. However, the clinical relevance of these microbiota-mediated effects in individuals with MetS remains to be confirmed.

### 5.6. Translational Validity of Preclinical Evidence

The preclinical studies summarized in Supplementary Tables S1–S5 differ from human MetS in several respects, limiting direct extrapolation. Only two in vivo studies used models explicitly designed to reproduce MetS, namely fructose-fed rats [57] and rats with MetS induced by a high-fat diet and a low dose of streptozotocin [86]. Most others used HFD-induced obesity, genetic models (ob/ob, TSOD, ApoE−/− or LDLR(+/−) animals), STZ-induced insulin deficiency [83], LPS challenge in piglets [125] or a lithogenic diet [98], each of which reproduce only part of the syndrome. In several studies, the preparation was given together with the obesogenic diet from the outset [75,81], so that prevention rather than reversal was tested, whereas fewer studies addressed established obesity or atherosclerosis [77,85,88]. Most in vivo studies included only male animals, group sizes were often small, down to three animals per group [81], and reduced food intake may have contributed to the weight changes reported in some studies [73,79].

In vitro effects were frequently observed at high concentrations, including 250–1000 μg/mL [90], up to 1000 μg/mL [70] or, for enzyme inhibition, up to 10 mg/mL [71] and 160 mg/mL [89], at which the inhibition of α-amylase did not exceed 10% [89]. Such concentrations are unlikely to reflect systemic exposure to high-molecular-weight polysaccharides, which largely escape digestion in the upper gastrointestinal tract and are fermented by the colonic microbiota [24,29]. For enzyme inhibition, intraluminal rather than plasma concentrations are the relevant comparator, which have not been determined after dietary intake. Molecular docking [55,89] predicts binding but does not establish target engagement, and in one study the docked compound itself was not tested experimentally [55]. Similarly, modulation of AMPK, PI3K/Akt, NF-κB, Nrf2 or PPAR signaling was typically inferred from changes in expression or phosphorylation at a single time point, which is consistent with, but does not demonstrate, a causal contribution to the metabolic phenotype.

Conversion of effective in vivo doses to human-equivalent doses (Table 2) indicates that several purified fractions and extracts were active at doses corresponding to approximately 0.5–4 g/day for a 60 kg adult, which is within the range used in human trials (0.54–10.4 g/day). Other effects were obtained at doses that would be impractical as supplements, such as about 19 g/day of insoluble fiber [82] or 29 g/day of mineral-enriched mycelial tablets [83], whereas isolated or synthetic compounds were active at milligram doses [77,108], a context more closely resembling drug development than dietary use. Preclinical findings obtained at plausible doses, in models reproducing several components of MetS and with chemically characterized preparations, such as those for *Trametes versicolor* heteropolysaccharides [86], represent the most suitable candidates for clinical testing.

### 5.7. Preparation Heterogeneity and Structure–Activity Considerations

The studies reviewed here investigated materials with fundamentally different compositions (Supplementary Tables S1–S5). For *G. lucidum* alone, the studies tested a spore oil standardized to 33.7% triterpenoids [66], sporoderm-broken spore powder, which has been reported to contain little triterpenoid material [162], spore and fruiting-body polysaccharides [74,98], polysaccharide peptides and a mycelial β-glucan [60,62,63], a proteoglycan [56], ethanolic extracts [55,73], aqueous preparations [57,64], a combination of fruiting-body extract and spores [58], triterpenoid-rich fractions [76,122] and isolated triterpenoids [119,120,121] and a synthetic meroterpene derivative [77]. Similarly, *C. militaris* was studied as fruiting-body powder, crude extract, isolated cordycepin and selenium-enriched or conventional polysaccharides [70,71,78,79,99,125], and the preparations reported as *C. sinensis* comprised an ethanolic extract, an exopolysaccharide from a cultured strain and a fermented product [72,123,124]. Effects reported for one preparation cannot be assumed to apply to another derived from the same species, and species-level statements in Section 4 should therefore be read as statements about the particular preparations tested.

For polysaccharides, biological activity depends on molecular weight, monosaccharide composition, glycosidic linkage type, degree of branching, chain conformation and solubility [25,26]. These parameters were reported inconsistently across studies. Molecular weight and monosaccharide composition were reported, for example, for *T. versicolor* heteropolysaccharides [86] and a *P. abieticola* polysaccharide [85], and the β-glucan content for the preparations used in several human trials [60,62,63,64,68], whereas the *P. ferulae* extract was characterized only by its total polysaccharide content determined colorimetrically [81], and in many other studies only the extraction solvent was specified. The available literature therefore does not permit a formal cross-study structure–activity analysis. Future studies should report, at a minimum, taxonomic authentication, the strain and fungal part used, the extraction procedure and yield, the content of marker compounds such as β-glucans, triterpenoids, cordycepin or ergothioneine and, for polysaccharides, the molecular weight distribution and monosaccharide composition.

### 5.8. Limitations of This Review

This review has several limitations. As a narrative review, it did not follow a prospectively registered systematic-review protocol, and no de novo quantitative synthesis was performed. Literature searches were conducted iteratively across thematic areas rather than through a single predefined systematic search and screening process. A complete record-level log was not maintained; therefore, reliable counts of records retrieved, deduplicated, screened and excluded at each stage cannot be reconstructed. Although the search concepts and the targeted PubMed strategy are provided in Supplementary Table S6, these limitations restrict the reproducibility and assessment of the comprehensiveness of the evidence identification process. Furthermore, the thematic, consensus-based study-selection procedure may have introduced selection bias. Therefore, gaps identified in the evidence map should be interpreted as gaps within the literature retrieved and included in this narrative review, rather than definitive evidence that no relevant studies exist.

Restricting the review to English-language publications may have excluded relevant studies, particularly those published in Asian-language journals. The methodological quality of primary studies was appraised descriptively rather than through an independent formal risk-of-bias assessment, although published risk-of-bias and GRADE evaluations were considered when interpreting the clinical evidence. Moreover, the substantial heterogeneity of fungal species, preparations, experimental models and outcomes precluded direct comparisons across studies. Finally, the evidence map summarizes the distribution and type of available studies rather than effect sizes or the certainty of individual findings and should not be interpreted as a measure of efficacy.

## 6. Conclusions and Perspectives

Current evidence indicates that edible and medicinal mushrooms contain diverse bioactive constituents that may influence biological processes relevant to MetS. However, the reported effects and proposed mechanisms are supported predominantly by preclinical studies, many of which used models, concentrations or doses with limited relevance to human MetS. Human evidence is dominated by *Ganoderma lucidum*. The only randomized trial in participants with diagnosed MetS that assessed its diagnostic components found no effect of *G. lucidum*, alone or combined with *Cordyceps sinensis*, on glycemia, blood pressure, lipids, waist circumference or MetS status. Systematic reviews likewise found no significant effects on fasting glucose, blood pressure or lipid profile, and the small pooled reduction in BMI, observed without a change in waist circumference and supported by very low-certainty evidence, does not indicate an improvement in MetS. Positive findings for single components, such as the triglyceride-lowering effect of a triterpene-rich spore oil, derive from single, unreplicated trials of specific preparations.

Interpretation is further limited by the heterogeneity of the preparations studied and their frequently inadequate chemical characterization. Among edible species with relatively consistent preclinical evidence, *Pleurotus ostreatus* and *Lentinula edodes* have been evaluated only in small trials with low-certainty or inconsistent results, whereas *Cordyceps militaris* and *Auricularia heimuer* have not been evaluated in trials assessing MetS components. Consequently, the available evidence neither establishes clinical efficacy nor supports species-specific dietary or clinical recommendations.

Future research should prioritize adequately powered, prospectively registered randomized controlled trials in populations meeting established diagnostic criteria for MetS, with intention-to-treat analyses and baseline-adjusted between-group comparisons. Studies should use taxonomically authenticated, chemically characterized and standardized preparations, with detailed reporting of the species or strain, fungal part, preparation method, chemical composition, dose, bioavailability and safety. Outcomes should include the MetS diagnostic components defined by the harmonized criteria, including waist circumference rather than BMI alone, and, where appropriate, MetS status as a composite outcome. Priority should be given to preparations that have shown activity in models reproducing several components of MetS at doses achievable in humans. Clinical research should also extend beyond *G. lucidum* to edible species with promising preclinical evidence but no or limited clinical evaluation. Markers of inflammation, oxidative stress, endothelial function, gut microbiota and mitochondrial function may be included as secondary mechanistic outcomes.

Edible and medicinal mushrooms therefore warrant further investigation as dietary sources of bioactive compounds with potential relevance to metabolic health. At present, however, the available human evidence remains insufficient to support species-specific dietary or clinical recommendations.

## Figures and Tables

**Table 1:** Human evidence on edible and medicinal mushrooms in relation to metabolic syndrome (MetS), classified by directness to MetS and by type of outcome. Diagnostic components follow the harmonized MetS definition [5].

Study	Species and Preparation (Dose, Duration)	Population (*n*)	Design and Key Methodological Features	Directness to MetS	MetS Diagnostic Components: Main Result	Related Outcomes: Main Result	Overlap with Systematic Reviews
Systematic reviews							
Klupp et al., 2015 [21]	*G. lucidum* (1.4–3 g/day in analyzed trials), 12–16 weeks	Adults with cardiovascular risk factors, 5 trials (*n* = 398); analyzed trials all in type 2 diabetes	Cochrane SR with MA; risk of bias low in one trial, unclear in four	Component-level	No significant effect on fasting glucose (single trial), BP or TG	No effect on HbA1c (WMD −0.10%, 95% CI −1.05 to 0.85), TC, LDL-C or BMI	Includes [58] as an unpublished trial
Dicks and Ellinger, 2020 [17]	*P. ostreatus*, mostly whole mushroom, various doses	Adults with or without cardiometabolic disease, 8 clinical trials	SR with RoB assessment, no MA; RoB mostly high or unclear	Component-level	Reductions in fasting or postprandial glucose and TG and, in some trials, BP; low certainty	Reductions in TC and LDL-C in some trials; body weight unchanged	–
Uffelman et al., 2023 [18]	Whole mushrooms, various species	Adults, RCTs and observational studies	SR, no MA	Component-level	Limited evidence for lower TG; no consistent effects on fasting glucose or BP	Limited evidence for lower hs-CRP; no consistent effects on other lipids or HbA1c	–
Alvianto et al., 2024 [59]	*G. lucidum*, mostly capsules	13 studies (7 in MA), including 2 classified as MetS populations	SR with MA	Component-level (MetS subgroup)	MetS subgroup: no difference in HDL-C or fasting glucose	MetS subgroup: no difference in LDL-C or TC	Includes [58]
Jafari et al., 2025 [19]	*G. lucidum* alone or in multi-ingredient products (6 of 17 trials), 200–11,200 mg/day, 1–24 weeks	Healthy, at-risk and chronically ill adults, 17 RCTs (*n* = 971)	SR with MA and GRADE, registered in PROSPERO, search to August 2024; most trials rated poor quality	Component-level	No effect on fasting glucose, BP, TG, HDL-C or waist circumference (WMD −0.45 cm, 95% CI −1.02 to 0.12); very low certainty	↓ BMI (WMD −0.43 kg/m^2^), ↓ creatinine (WMD −0.14 mg/dL, 6 trials, the pooled creatinine reduction was not statistically significant after separate exclusion of the trials by Wardhani et al. (2023) [60], Chen et al. (2023) [61], or Sargowo et al. (2022) [62]. No effect on body fat, inflammatory markers or liver enzymes	Includes [58,60,62,63,64]
Zadworny, 2025 [65]	*G. lucidum*, 720–5400 mg/day, 10 days–16 weeks	7 randomized or crossover trials	SR with RoB assessment (single reviewer), no MA	Component-level	No significant change in TG or HDL-C in any trial	No change in LDL-C; TC ↓ in 2 of 7 trials	Includes [58]
Primary trials							
Klupp et al., 2016 [58]	*G. lucidum* fruiting-body extract (10:1) with spores, 3 g/day, alone or with *C. sinensis* extract 1 g/day, 16 weeks	Adults with type 2 diabetes and MetS by NCEP-ATP III (*n* = 84; 54 active, 30 placebo)	DB, placebo-controlled, three-arm RCT, prospectively registered (ACTRN12606000485538); ITT (LOCF), baseline-adjusted ANCOVA; planned sample size not reached, active arms pooled	Direct	No effect on fasting glucose (0.03 mmol/L, 95% CI −0.90 to 0.96), BP, TG, HDL-C or waist circumference Number meeting MetS criteria not reduced	No effect on HbA1c (0.13%, 95% CI −0.35 to 0.60), BMI, TC, LDL-C, apolipoproteins or CRP No excess of adverse events	In [19,21,59,65]
Wang et al., 2025 [66]	*G. lucidum* spore oil (33.7% triterpenoids), 3.0 g/day (≈1.0 g triterpenes), 12 weeks	Adults aged 18–65 years with TG 1.70–2.25 mmol/L (*n* = 113 * randomized, 110 analyzed)	DB, placebo-controlled RCT, parallel groups (described as crossover in the title); registration not reported; per-protocol, end-of-treatment comparisons	Component-level	↓ TG, ↑ HDL-C vs. placebo	↓ TC, ↓ LDL-C, improved liver function tests; no change in renal markers	None (published after the search period of [19])
Wardhani et al., 2023 [60]	*G. lucidum* polysaccharide peptide (GLPP) 750 mg/day (540 mg β-1,3/1,6-D-glucan), 60 days	Adults with cardiometabolic syndrome by NCEP-ATP III (*n* = 62; 31/31)	DB, placebo-controlled RCT; registration not reported; only within-group comparisons reported	Indirect (MetS population, related outcomes only)	Not assessed	TNF-α and hs-CRP ↓ in both groups; ↓ MDA, ↑ SOD within GLPP group Urea and creatinine ↑ and eGFR ↓ within placebo group, no significant change within GLPP group	In [19]
Sargowo et al., 2022 [62]	β-1,3/1,6-D-glucan from *G. lucidum* mycelia, 540 mg/day, 3 months	Adults with obesity defined by BMI ≥ 25 kg/m^2^ (*n* = 68; 34/34)	Placebo-controlled RCT, blinding not described; registered (NCT05079529)	Indirect	Measured at baseline only	No between-group differences in urea, creatinine, nitric oxide, TNF-α or MDA; TNF-α and nitric oxide ↓ in both groups	In [19]
Rizal et al., 2020 [63]	GLPP 750 mg/day (540 mg β-glucan), 90 days	Adults with paroxysmal atrial fibrillation (*n* = 38; 20/18)	Randomized, placebo-controlled, "closed-label" (blinding not described); registration not reported	Component-level (BP assessed)	Greater ↓ SBP vs. placebo (−22.5 vs. −10.5 mmHg); no between-group difference in DBP	Greater ↓ MDA, hs-CRP, TNF-α, IL-1β and IL-6 vs. placebo; no change in BMI	In [19]
Iser-Bem et al., 2024 [64]	*G. lucidum* fruiting-body water extract (52.8% β-glucans), 2000 mg/day, 8 weeks	Women aged 60–80 years without neurodegenerative disease (*n* = 60 enrolled, 39 analyzed; 23/16)	DB, placebo-controlled; allocation by matching, randomization not reported; 35% attrition, per-protocol	Component-level (waist circumference assessed)	No difference in waist circumference	No difference in BMI or body fat ↓ Th17 cells, ↑ FOXP3, TGF-β and IL-10 gene expression vs. placebo	In [19]
Jung et al., 2019 [67]	*Paecilomyces hepiali* mycelial culture extract CBG-CS-2 (32% polysaccharides, 0.001% cordycepin), 1.68 g/day, 8 weeks	Healthy adults aged 20–75 years (*n* = 80 randomized, 79 analyzed)	DB, placebo-controlled RCT; retrospectively registered (NCT02814617); per-protocol	Indirect	Assessed as safety parameters; numerical results not reported	↑ NK-cell activity vs. placebo; no between-group differences in cytokines	None
Morales et al., 2021 [68]	*Lentinula edodes* β-glucan-enriched extract (composition reported), 10.4 g/day (3.5 g β-glucans), 8 weeks	Adults aged 18–65 years with untreated mild hypercholesterolemia (*n* = 57 randomized, 52 analyzed)	DB, placebo-controlled, parallel RCT; registered (NCT03550287); planned sample size not reached	Component-level	No differences vs. placebo in TG, HDL-C, fasting glucose, BP or waist circumference	No differences in TC, LDL-C, insulin, HbA1c, BMI, IL-1β, IL-6, TNF-α, oxLDL or CRP	None
Spim et al., 2021 [69]	Cereal bars containing dried *L. edodes*, 66 days	Adults with borderline-high TC, LDL-C or TG (*n* = 68; 36/32)	DB, placebo-controlled RCT; registered (NCT04186780)	Component-level	↓ TG by 10% within the intervention group	↑ glutathione, ↓ lipid peroxidation; dermatitis in 10% of participants	None

Directness to MetS: direct, studies enrolling participants with diagnosed MetS and assessing its diagnostic components; component-level, studies assessing at least one MetS diagnostic component as an intervention outcome, regardless of the population’s baseline metabolic status; indirect, studies assessing only related non-diagnostic outcomes or reporting diagnostic components solely as baseline or safety measures. Abbreviations: ANCOVA, analysis of covariance; BMI, body mass index; BP, blood pressure; CI, confidence interval; CRP, C-reactive protein; DB, double-blind; DBP, diastolic blood pressure; eGFR, estimated glomerular filtration rate; FOXP3, forkhead box P3; GLPP, *Ganoderma lucidum* polysaccharide peptide; GRADE, Grading of Recommendations Assessment, Development and Evaluation; HbA1c, glycated hemoglobin; HDL-C, high-density lipoprotein cholesterol; hs-CRP, high-sensitivity C-reactive protein; IL, interleukin; ITT, intention-to-treat; LDL-C, low-density lipoprotein cholesterol; LOCF, last observation carried forward; MA, meta-analysis; MDA, malondialdehyde; NK, natural killer; oxLDL, oxidized LDL; RCT, randomized controlled trial; RoB, risk of bias; SBP, systolic blood pressure; SOD, superoxide dismutase; SR, systematic review; TC, total cholesterol; TG, triglycerides; TGF-β, transforming growth factor beta; Th17, T helper 17; TNF-α, tumor necrosis factor alpha; WMD, weighted mean difference. Symbols: ↑, increase; ↓, decrease. * Wang et al. [66]: The abstract reports 110 randomized participants, whereas the Results report 113 randomized (58 intervention, 55 placebo) and 110 analyzed after three withdrawals.

**Table 3:** Evidence map of edible and medicinal mushrooms by species or preparation, outcome and level of evidence, based on the studies summarized in Table 1 and Supplementary Tables S1–S5. Columns 2–5 correspond to MetS diagnostic components, assessed in preclinical studies through the closest available measures, and column 6 to related outcomes.

Species or Preparation Group	Glucose Metabolism and Insulin Resistance	Lipids	Adiposity	Blood Pressure	Related Outcomes: Inflammation, Oxidative Stress (IO) and Gut Microbiota (M)
*G. lucidum* (extracts, spore powder and oil, polysaccharides, proteoglycan, triterpenoids)	V, S [55]; A [56,57] H (0) [58] SR (0) [19,21,59]	A [98] (biliary), [75,76] H [66]; H (0) [58] SR (0) [19,21,59,65]	A [73,74,75,76] H (0) [58] SR: BMI ↓, waist (0) [19]	No preclinical data H [63]; H (0) [58] SR (0) [19,21]	IO: V [119,120,121,122]; A [75,122] H [60,63,64]; H (0) [62] SR (0) [19] M: A [74]
Other *Ganoderma*-derived compounds (resinacein S from *G. resinaceum*, synthetic GMD)	A [77]	A [77]	A [77,108]	–	IO: A [77] M: A [77]
*C. militaris* (fruiting bodies, extract, cordycepin, polysaccharides)	V [70]; V, A [71]; A [79]	V, A [99]; V, A [78]; A [79]	V, A [99]; A [79]	–	IO: A [125] M: A [125]
Preparations reported as *C. sinensis* or *O. sinensis*, including *Paecilomyces hepiali*	V [72] H (0) * [58]	H (0) * [58]	H (0) * [58]	H (0) * [58]	IO: V [123,124]; H (immune, *P. hepiali* mycelial extract) [67]
*C. guangdongensis* (multi-component formula)	A [80]	A [80]	A [80]	–	M: A [80]
*P. ostreatus*	V, S [89] (weak) A [88] SR (low certainty) [17]	A [82]; A (0) [100] SR (low certainty) [17]	A [88] SR: body weight (0) [17]	V (enzymes) [113] SR (some trials) [17]	M: A [88]
*P. citrinopileatus* + *H. erinaceus* (mineral-enriched tablets)	A [83] (STZ model)	A [83]	–	–	IO: A [83]
*P. ferulae*	–	A [81] (TC, LDL-C)	V, A [81]	–	–
*P. abieticola*	–	A [84]	–	–	IO: A [84,85]
*L. edodes*	V [90] H (0) [68]	V [90] (gene expression) H [69] (within-group TG reduction only); H (0) [68]	H (0) [68]	H (0) [68]	IO: H [69] (oxidative); H (0) [68] (cytokines) M: H [68] (composition)
*L. subnudus*	–	–	–	V (enzymes) [113]	–
*T. versicolor*	A [86] (MetS model)	A [86] (MetS model)	–	A [86] (MetS model)	IO: A [86]
*A. heimuer*	–	–	–	–	IO: A [87]
Whole edible mushrooms, various species	SR (0) [18]	SR: TG ↓ (limited), other lipids (0) [18]	–	SR (0) [18]	IO: SR: hs-CRP ↓ (limited) [18]

Abbreviations and symbols: A, animal study; IO, inflammation and oxidative stress; M, gut microbiota; GMD, ganomycin I-based meroterpene derivative; H, human intervention trial; S, in silico study; SR, systematic review; STZ, streptozotocin; V, in vitro study; (0), no significant effect; –, no data. Unless marked (0), entries indicate that a favorable effect was reported in at least one study. This designation does not imply replication, clinical significance, high certainty of evidence or comparability between preparations. Symbols: ↓, decrease. * Active arms with and without *C. sinensis* were pooled, so the effect of *C. sinensis* cannot be separated. The review in the last row also includes species not addressed in the preclinical synthesis.

## Data Availability

The data synthesized in this study are available within the article and its Supplementary Materials.

## Supplementary Materials

The following supporting information can be downloaded at https://www.mdpi.com/article/10.3390/nu18213646/s1, Table S1: Preclinical evidence on edible and medicinal mushrooms in relation to carbohydrate metabolism and insulin sensitivity [55,56,57,70,71,72,79,83,89,90]; Table S2: Preclinical evidence on edible and medicinal mushrooms in relation to lipid metabolism and dyslipidemia [78,79,82,90,98,99,100]; Table S3: Preclinical evidence on edible and medicinal mushrooms in relation to adiposity and obesity-related outcomes [73,74,75,76,77,79,80,81,88,99,108]; Table S4: Preclinical evidence on edible and medicinal mushrooms in relation to blood pressure regulation and vascular-related enzyme activity [86,113]; Table S5: Preclinical evidence on edible and medicinal mushrooms in relation to inflammation, oxidative stress and gut microbiota-related outcomes [75,84,85,87,119,120,121,122,123,124,125]; Table S6: Search concepts and targeted PubMed search strategy for human evidence.
