# Supplementary material for: Edible and Medicinal Mushrooms in Metabolic Syndrome: From Mechanistic Plausibility to Clinical Evidence—A Narrative Review
Source: Nutrients. 2026 Nov 2;18(21):3646. doi: 10.3390/nu18213646 (PMC13765278; doi:10.3390/nu18213646)
Supplement: Supplementary file 1 [file nutrients-18-03646-s001.zip › nutrients-4625409-supplementary.pdf]

Table S1. Preclinical evidence on edible and medicinal mushrooms in relation to carbohydrate metabolism and insulin sensitivity. Preparation types (whole material, crude extract, enriched fraction, isolated compound, synthetic derivative or multi-component formula) are given in square brackets in the Compound/Extract column [55–57,70–72, 79,83,89,90].

Species	Model/System	Study design	Compound/Extract	Dose/Concentration	Main outcomes	Reference
Ganoderma lucidum	PA-induced metabolic dysfunction and insulin resistance model in HepG2 cells	In vitro and in silico study	Ethanol extract of *G. lucidum* (GLAE) [crude extract]	20, 40, and 80 µg/mL; PA: 0.2 mM	In vitro: ↑ glucose consumption, ↑ GLUT2 membrane localization, ↓ TG/TC accumulation, ↓ mRNA of CASP3, PRKACA, CYP19A1, NR3C1 and JUN, ↑ PGR and RXRA (whole extract); *in silico*: predicted binding of β-sitosterol to CASP3, PRKACA and PGR (not experimentally validated f or the isolated compound.)	[55]
Ganoderma lucidum	Male Wistar rats with fructose-induced metabolic syndrome	Animal study	Aqueous suspension of lyophilized *G. lucidum* mycelium extract [crude extract]	1 g/kg body weight for 14 days	↓ AUCglu after glucose loading, ↓ fructosamine, normalization of starch tolerance, inhibition of α-amylase and α-glucosidase activity in vitro	[57]
Ganoderma lucidum	Male C57BL/6 WT mice and ob/ob mice, WT n = 10, ob/ob n = 50, 5 groups, n = 10/group, L6 rat skeletal muscle cells	In vivo and in vitro study	Fudan-Yueyang *G. lucidum* proteoglycan (FYGL) [enriched fraction]	In vivo: 150, 300, and 400 mg/kg/day for 4 weeks; in vitro: 0–150 µg/mL in L6 cells	↓ fasting blood glucose, ↓ insulin resistance index, ↑ glucose uptake in L6 cells, modulation of IRS1/PI3K/Akt/AMPK/GLUT4 signaling pathway	[56]
Cordyceps militaris	Insulin-resistant HepG2 cells	In vitro	Selenium-enriched *C. militaris* polysaccharides obtained by biotransformation (Se-CMP) [enriched fraction]	100-1000 µg/mL, 1000 µg/mL used for mechanistic analyses	↑ glucose consumption, ↑ glycogen synthesis, ↓ ROS, ↑ SOD, CAT and GSH-Px activities, activation of PI3K/AKT/GLUT4 pathway	[70]
Cordyceps militaris	Enzyme assays and male C57BL/6J diabetic mice, Con n = 5, diabetes group n = 25; after diabetes induction: Dia, Met, and CM-P groups, n = 5/group	In vitro and animal study	Polysaccharides isolated from *C. militaris* fruiting bodies (CM-P) [enriched fraction]	In vitro: 1.0-10.0 mg/mL, in vivo: 200, 400, and 800 mg/kg/day for 4 weeks	Inhibition of α-amylase and α-glucosidase, ↓ blood glucose and insulin levels, improved insulin sensitivity and glucose tolerance, ↓ oxidative stress and inflammation	[71]
Cordyceps militaris	Male C57BL/6J mice, HFD-induced obesity	Animal study	Dried fruiting-body powder of conventional	250 mg/kg/day for 12 weeks	↓ fasting blood glucose, improved oral glucose	[79]

	model (n = 32; 4 groups, n = 8/group)		and selenium-enriched *C. militaris* [whole material]		tolerance, no difference between conventional and selenium-enriched material	
Pleurotus ostreatus	α-amylase, α-glucosidase and PEPCK enzyme assays	In vitro and in silico study	Methanolic extract of *P. ostreatus* (MEPO) [crude extract, composition by GC-FID]	32–160 mg/mL (as reported)	Weak inhibition of α-amylase (max. 10.0%) and α-glucosidase (max. 43.4%) at 160 mg/mL, IC50 1074.05 and 246.58 mg/mL (extrapolated beyond the tested range), docking: rutin and naringin interacted with α-amylase, α-glucosidase and PEPCK	[89]
Pleurotus citrinopileatus, *Hericium erinaceus*	Male KM mice with STZ-induced diabetes, n = 40, 5 groups, n = 8/group	Animal study	Buccal tablets prepared from *P. citrinopileatus* and *H. erinaceus* mycelia enriched with Cr, Zn and Ge [whole material with added minerals]	Low dose: 2 g/kg; high dose: 6 g/kg for 3 weeks; gavage volume: 0.1 mL/10 g body weight	↓ blood glucose (29.1% relative to pre-administration in the high-dose group),↓ TC, TG and LDL-C, ↑ HDL-C; ↑ CAT and SOD activities; mod ulation of gut microbiota composit ion	[83]
Lentinula edodes (shiitake)	L6 myoblasts/myotubes	In vitro	Fruiting body extracts, water, ethanol, methanol and hydroalcoholic extracts 50:50, 75:25, 25:75 [crude extracts]	250, 500, and 1000 μg/mL	↑ glucose uptake, ↑ GLUT4 expression/translocation, ↑ p-AMPK, hydroalcoholic extracts 50:50 and 75:25 showed the strongest metabolic activity	[90]
Preparation reported as *Cordyceps sinensis* (fermented)	HK-2 human renal proximal tubular cells exposed to high glucose (30 mM)	In vitro and in silico (network pharmacology) study	Fermented *C. sinensis* [crude preparation]	50 μg/mL for 48 h	↓ cytotoxicity and apoptosis, ↑ proliferation, ↓ Bax, caspase-3, VEGFA, p-AKT and p-ERK, ↑ PTEN	[72]

AUCglu, area under the glucose curve; CAT, catalase; FYGL, Fudan-Yueyang *G. lucidum* proteoglycan; GLAE, *G. lucidum* ethanol extract; GSH-Px, glutathione peroxidase; HFD, high-fat diet; IC50, half-maximal inhibitory concentration; MEPO, methanolic extract of *P. ostreatus*; PA, palmitic acid; PEPCK, phosphoenolpyruvate carboxykinase; ROS, reactive oxygen species; Se-CMP, selenium-enriched *C. militaris* polysaccharides; SOD, superoxide dismutase; STZ, streptozotocin; TC, total cholesterol; TG, triglycerides.

Table S2. Preclinical evidence on edible and medicinal mushrooms in relation to lipid metabolism and dyslipidemia. Preparation types (whole material, crude extract, enriched fraction, isolated compound, synthetic derivative or multi-component formula) are given in square brackets in the Compound/Extract column [78,79,82,90,98–100].

Species	Model/System	Study design	Compound/Extract	Dose/Concentration	Main outcomes	Reference
Ganoderma lucidum	Male C57BL/6J mice, lithogenic diet-induced cholesterol gallstone model (n = 40, 4 groups, n = 10/group)	Animal study	*G. lucidum* polysaccharide (GLP) *[enriched fraction]*	Lithogenic diet supplemented with 2% or 4% GLP for 6 weeks	↓ cholesterol gallstone formation, ↓ total cholesterol in gallbladder bile, ↓ cholesterol saturation index	[98]

Cordyceps militaris	3T3-L1 adipocytes and male TSOD mice, a model of obese type 2 diabetes (n = 5/group)	In vitro and animal study	*C. militaris* extract (CM) [crude extract] and cordycepin [isolated compound]	CM: 50 μg/mL in vitro and 0.1% diet in vivo, cordycepin: 5-10 μg/mL in vitro and 0.000357% diet in vivo for 3 weeks	↓ lipid droplet accumulation, ↑ HSL-related lipolysis, ↓ LDL/VLDL cholesterol	[99]
Cordyceps militaris	3T3-L1 preadipocytes and male LDLR (+/−) hamsters with hyperlipidemia-like lipid profile (n = 20, 4 groups, n = 5/group)	In vitro and animal study	Polysaccharide CM1 isolated from *C. militaris* [enriched fraction]	In vitro: 100 μg/mL; *in vivo*: 100 mg/kg/day for 5 months	↓ plasma TC, ↓ plasma TG, ↓ epididymal fat index, ↓ apolipoprotein B48, inhibition of preadipocyte differentiation, modulation of genes and proteins involved in lipid metabolism	[78]
Cordyceps militaris	Male C57BL/6J mice, HFD-induced obesity model (n = 32; 4 groups, n = 8/group)	Animal study	Dried fruiting-body powder of conventional and selenium-enriched *C. militaris* [whole material]	250 mg/kg/day for 12 weeks	↓ serum TG, TC and LDL-C, no difference between conventional and selenium-enriched material	[79]
Pleurotus ostreatus	Male SD rats, HFD-induced obesity model (n = 8/group after obesity induction)	Animal study	Insoluble dietary fiber isolated from dried fruiting bodies of *P. ostreatus* (POIDF) [enriched fraction]	1, 1.5 or 2 g/kg/day for 6 weeks	↓ TG, ↓ LDL-C, ↓ NEFA, ↓ TC in selected groups, ↓ body weight and fat accumulation	[82]
Pleurotus ostreatus	Male Wistar rats with prednisolone-induced hyperlipidemia (n = 20, 5 groups, n = 4/group)	Animal study	Dried powdered *P. ostreatus* as dietary supplement [whole material]	10 g/day and 20 g/day for 4 weeks	Non-significant ↓ TC, TG, LDL and VLDL compared with negative control ($p > 0.05$)	[100]
Lentinula edodes	L6 myoblasts/myotubes	In vitro study	Fruiting body extracts of *L. edodes*: water, ethanolic, methanolic and hydroalcoholic extracts [crude extracts]	250, 500 and 1000 μg/mL, hydroalcoholic extracts 50:50, 75:25 and 25:75	↑ p-AMPK, differential regulation of PPARγ and FASN, suggesting interaction with lipid metabolism	[90]

CM, *C. militaris* extract; GLP, *G. lucidum* polysaccharide; HFD, high-fat diet; HSL, hormone-sensitive lipase; LDL-C, low-density lipoprotein cholesterol; NEFA, non-esterified fatty acids; POIDF, *P. ostreatus* insoluble dietary fiber; TC, total cholesterol; TG, triglycerides; TSOD, Tsumura Suzuki obese diabetes; VLDL, very-low-density lipoprotein.

Table S3. Preclinical evidence on edible and medicinal mushrooms in relation to adiposity and obesity-related outcomes. Preparation types (whole material, crude extract, enriched fraction, isolated compound, synthetic derivative or multi-component formula) are given in square brackets in the Compound/Extract column [73–77,79–81,88,99,108].

Species	Model/System	Study design	Compound/Extract	Dose/Concent ration	Main outcomes	Reference

Ganoderma lucidum	Swiss mice, both sexes (n = 40; 4 groups, n = 10/group)	Animal study	*G. lucidum* ethanol extract [crude extract]	100, 200 or 400 mg/kg per day for 30 days	↓ body weight gain (100 and 200 mg/kg), ↓ feed intake	[73]
Ganoderma lucidum	Male C57BL/6J mice, HFD-induced obesity model (n = 36; 6 groups, n = 6/group)	Animal study	Polysaccharide extracted from sporoderm-broken spores of *G. lucidum* (BSGLP) [enriched fraction]	100 mg/kg or 300 mg/kg for 12 weeks	↓ body weight gain, ↓ cell diameter of adipocytes, ↓ fat accumulation and steatosis in liver	[74]
Ganoderma -derived compound	Male ApoE−/− mice fed a HFD for 8 weeks before treatment (n = 33; 5 groups including atorvastatin control, n = 5–8/group)	Animal study	Synthetic meroterpene derivative based on ganomycin I isolated from *Ganoderma* spp. (GMD) [synthetic derivative]	5 or 10 mg/kg/day by oral gavage for 9 weeks	↓ body weight gain, insulin resistance, serum lipids and atherosclerotic plaque area, ↑ intestinal *Parabacteroides merdae*	[77]
Ganoderma lucidum	Male C57BL/6J mice, HFD-induced obesity model (n = 40; 4 groups including L-carnitine control, n = 10/group)	Animal study	Sporoderm-broken spore powder of *G. lucidum* (SSPL) suspended in distilled water [whole material]	650 mg/kg/day by oral gavage (100 μL) for 12 weeks	↓ body weight gain, ↓ liver weight, ↓ epididymal fat weight, ↓ adipocyte size, ↓ serum TC, TG and LDL-C, ↑ HDL-C, food intake unchanged	[75]
Ganoderma lucidum	Male C57BL/6J mice with HFD-induced obesity (n = 36; 4 groups, n = 9/group) and 3T3-L1 preadipocytes	In vitro and animal study	*G. lucidum* triterpenoids (GLT) [enriched fraction]	*In vivo*: 100 or 200 mg/kg/day by oral gavage for 12 weeks; in vitro: 50–400 μg/mL during the initial 2 days of 3T3-L1 differentiation	↓ body weight gain, serum TG and TC, eWAT accumulation and adipocyte size; inhibition of preadipocyte differentiation and lipid accumulation via PRKCQ-related signaling	[76]
Ganoderma resinaceum	Male C57BL/6 mice (n = 24; 4 groups, n = 6/group)	Animal study	Resinacein S, a triterpenoid extracted from *G. resinaceum* [isolated compound]	Dose not inde pendently veri fied	↓ body weight gain, ↑ thermogenesis and energy expenditure	[108]
Cordyceps militaris	3T3-L1 mouse preadipocytes and male TSOD mice (n = 5/group)	In vitro and in vivo study	*C. militaris* extract (CM) [crude extract] and cordycepin [isolated compound]	CM: 50 μg/mL (in vitro), 0.1% diet (in vivo); cordycepin: 5-10 μg/mL (in vitro), 0.000357% diet (*in vivo*) for 3 weeks	↓ preadipocyte differentiation and lipid accumulation ↑ lipolysis	[99]

Cordyceps militaris	Male C57BL/6J mice (n = 32; 4 groups, n = 8/group)	Animal study	Dried fruiting-body powder of conventional and selenium-enriched *C. militaris* [whole material]	250 mg/ kg per day for 12 weeks	↓ body weight gain, ↓ liver weight and epididymal, perirenal and inguinal fat mass, ↓ energy intake, no difference between conventional and selenium-enriched material	[79]
Cordyceps guangdongensis	Male SPF C57BL/6J mice (n = 45; 3 groups, n = 15/group)	Animal study	Multi-component lipid-lowering formula containing powdered fruiting bodies of *C. guangdongensis* (CGLC) [multi-component formula]	1.5 g/kg per day for 14 weeks	↓ body weight, ↓ fat accumulation, ↓ hepatic lipid droplet accumulation, improved glucose tolerance and lipid profile, ↑ short-chain fatty acids, modulation of gut microbiota	[80]
Pleurotus ferulae	3T3-L1 adipocytes and male C57BL/6 mice, HFD-induced obesity model (n = 3/group as reported in figure legends)	In vitro and animal study	Hot water extract (1:20) of *P. ferulae* (PWE) [crude extract, total polysaccharide content determined colorimetrically]	*In vivo*: 100 mg/kg/day in drinking water for 8 weeks; in vitro: 100 and 200 μg/mL	↓ body weight gain, ↓ fat mass, ↓ adipocyte size, ↓ serum TC and LDL-C, ↓ lipid accumulation and ↓ PPARγ and C/EBPα in 3T3-L1 cells	[81]
Pleurotus ostreatus	Male C57BL/6J mice with obesity established by 6 weeks of HFD (n = 40; 5 groups, n = 8/group)	Animal study	Dried powdered fruiting bodies of *P. ostreatus* incorporated into the HFD [whole material]	2.5%, 5% and 10% (w/w in HFD) for a further 6 weeks	↓ body weight gain, ↓ white adipose tissue, maintained glucose homeostasis, gut microbiota shifted towards that of lean mice	[88]

In the *P. ferulae* study [81], the ethanolic extract (PEE) was compared with PWE only in a preliminary 3T3-L1 lipid-accumulation screen reported in the supplementary material of the original article, in which PWE was more effective, and PEE was not tested *in vivo*. ApoE−/−, apolipoprotein E-deficient; BSGLP, polysaccharide from sporoderm-broken spores of *G. lucidum*; C/EBPα, CCAAT/enhancer-binding protein α; CGLC, *C. guangdongensis* lipid-lowering formula; eWAT, epididymal white adipose tissue; GLT, *G. lucidum* triterpenoids; GMD, ganomycin I-based meroterpene derivative; HDL-C, high-density lipoprotein cholesterol; HFD, high-fat diet; LDL-C, low-density lipoprotein cholesterol; PPARγ, peroxisome proliferator-activated receptor γ; PWE, *P. ferulae* water extract; SSPL, sporoderm-broken spore powder of *G. lucidum*; TC, total cholesterol; TG, triglycerides; TSOD, Tsumura Suzuki obese diabetes.

Table S4. Preclinical evidence on edible and medicinal mushrooms in relation to blood pressure regulation and vascular-related enzyme activity. Preparation types (whole material, crude extract, enriched fraction, isolated compound, synthetic derivative or multi-component formula) are given in square brackets in the Compound/Extract column [86,113].

Species	Model/System	Study design	Compound/Extract	Dose/Concentration	Main outcomes	Reference
Pleurotus ostreatus	Lung and heart homogenates from male Wistar rats (n=10)	In vitro study	Aqueous extract of *P. ostreatus* prepared from dried powdered sample [crude extract]	1-25 μg/mL, depending on assay	↓ ACE, arginase, AChE and ADA activity, with stronger inhibition of ACE and arginase than *L. subnudus*	[113]
Lentinus subnudus	Lung and heart homogenates from male Wistar rats (n=10)	In vitro study	Aqueous extract of *L. subnudus* prepared from dried powdered sample [crude extract]	1-25 μg/mL, depending on assay	↓ ACE, arginase, AChE and ADA activity, with stronger inhibition of cholinesterase than *P. ostreatus*	[113]
Trametes versicolor	Wistar rats with MetS induced by a 4-week HFD followed by a single low dose of STZ (25 mg/kg) (n = 40; 5 groups, n = 8/group)	Animal study	Trametes versicolor heteropolysaccharides (TVH) [enriched fraction, characterized by FT-IR, GC-MS monosaccharide analysis and HPLC, Mw ≈ 2.6 kDa]	100, 200 or 300 mg/kg/day orally for 4 weeks	↓ systolic and diastolic blood pressure at all doses (without dose dependence), improved glucose tolerance and ↓ insulin at the highest dose, ↓ TG and ↑ HDL-C, improved redox status and cardiac function *ex vivo*	[86]

ACE, angiotensin-converting enzyme; AChE, acetylcholinesterase; ADA, adenosine deaminase; FT-IR, Fourier-transform infrared spectroscopy; GC-MS, gas chromatography–mass spectrometry; HDL-C, high-density lipoprotein cholesterol; HFD, high-fat diet; HPLC, high-performance liquid chromatography; MetS, metabolic syndrome; Mw, molecular weight; STZ, streptozotocin; TG, triglycerides; TVH, *T. versicolor* heteropolysaccharides.

Table S5. Preclinical evidence on edible and medicinal mushrooms in relation to inflammation, oxidative stress and gut microbiota-related outcomes. Preparation types (whole material, crude extract, enriched fraction, isolated compound, synthetic derivative or multi-component formula) are given in square brackets in the Compound/Extract column [75,84,85,87,119–125].

Species	Model/System	Study design	Compound/Extract	Dose/Concentration	Main outcomes	Reference
Ganoderma lucidum	RAW264.7 macrophages	In vitro study	Lucidone D (LUC) [isolated compound]	10, 20, and 40 μM; LPS: 1 μg/mL	↓ NO, TNF-α and IL-6 levels, ↓ COX-2 and iNOS expression	[121]
Ganoderma lucidum	HUVECs exposed to disturbed flow and mouse model of partial carotid artery ligation	In vitro and animal study	Ganoderma triterpenoids (GT) [enriched fraction]	In vitro: 500 μg/mL, LSS 12 dyn/cm², OSS ±5 dyn/cm² or static control for 24 h; *in vivo*: oral *G. lucidum* (300	↓ VCAM-1, TNF-α and IL-6 expression in vitro, prevention of neointimal thickening *in vivo*	[122]

				mg/kg/day) or subcutaneous GT		
Ganoderma lucidum	BV-2 microglial cells	In vitro study	Ganoderterpene A [isolated compound]	25 μM; LPS: 1 μg/mL	↓ iNOS, TNF-α, COX-2, IL-1β and IL-6 expression	[119]
Ganoderma lucidum	Human nucleus pulposus cells isolated from patients with intervertebral disc degeneration	In vitro study	Ganoderic acid A (GAA) [isolated compound]	6.25, 12.5, and 25 μM; IL-1β: 10 ng/mL	↓ NO, PGE2, TNF-α and IL-6 levels, ↓ COX-2 and iNOS expression	[120]
Ganoderma lucidum	Male C57BL/6J mice, HFD-induced obesity model (n = 40; 4 groups, n = 10/group)	Animal study	Sporoderm-broken spore powder of *G. lucidum* (SSPL) [whole material]	650 mg/kg/day by oral gavage for 12 weeks	↓ expression of inflammation-related genes TNF-α, IL-6 and IL-1β	[75]
Cordyceps sinensis	LPS-induced THP-1 macrophages and BEAS-2B bronchial epithelial cells in a transwell co-culture system	In vitro study	Cordyceps sinensis extract (CSE) [crude extract]	0.1–100 μg/mL	↓ IL-6, TNF-α, IL-1β and IL-8, activation of Nrf2/HO-1, modulation of TLR and NLR signaling	[123]
Preparation reported as *Cordyceps sinensis* (cultured strain Cs-HK1)	RAW264.7 macrophages	In vitro study	Exopolysaccharide EPS-LM from Cs-HK1 mycelial fermentation [enriched fraction]	25, 50, 100, and 200 μg/mL; LPS-induced inflammation	↓ NO and ROS production, ↓ TLR4 and MyD88 expression, inhibition of TLR4/MyD88/NF-κB signaling	[124]
Cordyceps militaris	Healthy male weaned piglets exposed to LPS (n = 24; 4 groups, n = 6/group)	Animal study	*C. militaris* extract (CME) [crude extract] and cordycepin (CPN) [isolated compound]	CME- or CPN-supplemented feed providing 1 mg/kg cordycepin for 21 days; LPS: 100 μg/kg body weight	CPN reversed the LPS-induced decrease in serum IL-10 but increased serum TNF-α; CPN and CME ↓ ileal IL-6, IL-8, TNF-α and IL-10, attenuated oxidative stress and modulated the gut microbiota; CPN prevented the LPS-associated decrease in ileal butyrate	[125]
Pleurotus abieticola	Male C57BL/6J mice, HFD-induced hyperlipidemia model (n = 32; 4	Animal study	Neutral polysaccharide isolated from *P. abieticola* fruiting	100 mg/kg per day for 4 weeks	↓ hepatic ROS, ↓ TNF-α, IL-1β, IL-6, IL-17A, IL-22 and IL-23, ↑ IL-10,	[84]

	groups, n = 8/group)		bodies (PAPS1) [enriched fraction]		inhibition of NF-κB/STAT3 signaling	
Pleurotus abieticola	Male ApoE−/− mice fed a HFD for 8 weeks before treatment (n = 18; 3 groups including rosuvastatin control, n = 6/group) and C57BL/6J controls (n = 6)	Animal study	Neutral polysaccharide PAPS2 isolated from *P. abieticola* fruiting bodies [enriched fraction, Mw ≈ 19.6 kDa, structure characterized by NMR]	100 mg/kg/day orally for 4 weeks	↑ SOD and GSH-Px, ↑ Nrf2, inhibition of TLR4/NF-κB signaling	[85]
Auricularia heimuer	Male ApoE−/− mice, HFD-induced atherosclerosis model (n = 24; 3 groups, n = 8/group)	Animal study	*A. heimuer* preparation *[preparation type not specified]*	500 mg/kg per day for 4 weeks	↓ ROS in vascular endothelium, ↑ Nrf2, HO-1, SOD-1 and IL-10, ↓ phosphorylation of NF-κB, IκBα and IKKα/β, ↓ IL-6 and LTB4R2	[87]

Some studies used non-metabolic inflammation or oxidative stress models. Their findings are presented as mechanistic evidence and should not be interpreted as direct evidence of efficacy in metabolic syndrome. ApoE−/−, apolipoprotein E-deficient; COX-2, cyclooxygenase-2; CME, *C. militaris* extract; CPN, cordycepin; CSE, *C. sinensis* extract; GAA, ganoderic acid A; GSH-Px, glutathione peroxidase; GT, *Ganoderma* triterpenoids; HFD, high-fat diet; HO-1, heme oxygenase-1; HUVECs, human umbilical vein endothelial cells; IKK, IκB kinase; iNOS, inducible nitric oxide synthase; LPS, lipopolysaccharide; LSS, laminar shear stress; LTB4R2, leukotriene B4 receptor 2; LUC, lucidone D; NLR, NOD-like receptor; NMR, nuclear magnetic resonance; Nrf2, nuclear factor erythroid 2-related factor 2; OSS, oscillatory shear stress; PAPS, *P. abieticola* polysaccharide; ROS, reactive oxygen species; SOD, superoxide dismutase; SSPL, sporoderm-broken spore powder of *G. lucidum*; STAT3, signal transducer and activator of transcription 3; TLR, Toll-like receptor; VCAM-1, vascular cell adhesion molecule-1.

Table S6. Search concepts and targeted PubMed search strategy for human evidence.

(a) Search term groups

Concept group	Search terms (title and abstract)
Group 1: Mushrooms and fungal compounds	mushroom, edible mushroom, medicinal mushroom, *Ganoderma*, reishi, lingzhi, *Cordyceps*, *Ophiocordyceps*, cordycepin, *Pleurotus*, oyster mushroom, *Lentinula*, shiitake, *Hericium*, *Auricularia*, *Trametes*, β-glucan, ganoderic acid, mushroom polysaccharide
Group 2: Metabolic syndrome and its components	metabolic syndrome, glucose metabolism, insulin resistance, type 2 diabetes, dyslipidemia, hyperlipidemia, obesity, blood pressure, hypertension
Group 3: Related mechanisms	inflammation, oxidative stress, gut microbiota
Combination and limits	The groups summarize the concepts used in the iterative thematic searches and do not represent a single historical database-specific query. Searches covered English-language publications from database inception through 10 April 2026.

(b) Targeted PubMed search for human evidence (search date: 10 April 2026)

Step	Concept	Search string
#1	Mushrooms and fungal taxa	mushroom*[tiab] OR Ganoderma[tiab] OR reishi[tiab] OR lingzhi[tiab] OR Cordyceps[tiab] OR Ophiocordyceps[tiab] OR cordycepin[tiab] OR Pleurotus[tiab] OR "oyster mushroom"[tiab] OR Lentinula[tiab] OR shiitake[tiab] OR Hericium[tiab] OR Auricularia[tiab] OR Trametes[tiab]
#2	Metabolic syndrome and its components	"metabolic syndrome"[tiab] OR "insulin resistance"[tiab] OR glucose[tiab] OR diabetes[tiab] OR dyslipidemia[tiab] OR hyperlipidemia[tiab] OR cholesterol[tiab] OR triglyceride*[tiab] OR obesity[tiab] OR "blood pressure"[tiab] OR hypertension[tiab]
#3	Study type	randomized controlled trial[pt] OR clinical trial[pt] OR systematic review[pt] OR meta-analysis[pt]
#4	Final search	#1 AND #2 AND #3 AND english[la] AND ("1900/01/01"[dp] : "2026/04/10"[dp])

Part (b) presents the documented targeted PubMed strategy for human evidence. The complete historical Embase-specific queries were not retained and cannot be reliably reconstructed.
